# Optimized Real-Time Biomimetic Neural Network on FPGA for Bio-hybridization

**DOI:** 10.3389/fnins.2019.00377

**Published:** 2019-04-24

**Authors:** Farad Khoyratee, Filippo Grassia, Sylvain Saïghi, Timothée Levi

**Affiliations:** ^1^Laboratoire de l'Intégration du Matériau au Système, Bordeaux INP, CNRS UMR 5218, University of Bordeaux, Talence, France; ^2^LTI Laboratory, EA 3899, University of Picardie Jules Verne, Amiens, France; ^3^Institute of Industrial Science, The University of Tokyo, Tokyo, Japan

**Keywords:** Hodgkin-Huxley, neuromorphic, biomimetic, neural diseases, spiking neural network, bio-hybrid

## Abstract

Neurological diseases can be studied by performing bio-hybrid experiments using a real-time biomimetic Spiking Neural Network (SNN) platform. The Hodgkin-Huxley model offers a set of equations including biophysical parameters which can serve as a base to represent different classes of neurons and affected cells. Also, connecting the artificial neurons to the biological cells would allow us to understand the effect of the SNN stimulation using different parameters on nerve cells. Thus, designing a real-time SNN could useful for the study of simulations of some part of the brain. Here, we present a different approach to optimize the Hodgkin-Huxley equations adapted for Field Programmable Gate Array (FPGA) implementation. The equations of the conductance have been unified to allow the use of same functions with different parameters for all ionic channels. The low resources and high-speed implementation also include features, such as synaptic noise using the Ornstein–Uhlenbeck process and different synapse receptors including AMPA, GABAa, GABAb, and NMDA receptors. The platform allows real-time modification of the neuron parameters and can output different cortical neuron families like Fast Spiking (FS), Regular Spiking (RS), Intrinsically Bursting (IB), and Low Threshold Spiking (LTS) neurons using a Digital to Analog Converter (DAC). Gaussian distribution of the synaptic noise highlights similarities with the biological noise. Also, cross-correlation between the implementation and the model shows strong correlations, and bifurcation analysis reproduces similar behavior compared to the original Hodgkin-Huxley model. The implementation of one core of calculation uses 3% of resources of the FPGA and computes in real-time 500 neurons with 25,000 synapses and synaptic noise which can be scaled up to 15,000 using all resources. This is the first step toward neuromorphic system which can be used for the simulation of bio-hybridization and for the study of neurological disorders or the advanced research on neuroprosthesis to regain lost function.

## Introduction

Brain disorders are among the leading causes of disabilities worldwide. The increasing number of neurological diseases raised scientists to reconsider the way of studying the human cells and the process of healing brain afflictions. Along with advanced technology, the combination of technological devices and biological neurons, also called hybrid neuromorphic engineering, was explored to find advanced solutions by designing bio-hybrid devices. Reproducing neuro-mimetic activities and improving the connection between living cells and machines became mandatory for designing neuroprosthesis. Such devices (Nicolelis and Lebedev, 2009; Hochberg et al., 2012; Bonifazi et al., 2013) have been focused on the interactions with neuronal cell assemblies especially on mimicking the spontaneous activities of the biological neural networks. To perform in the future replacing experiments (Jung et al., 2001), meaning that damaged biological neural network will be replaced by artificial neural network, detailed models of neurons and synapses are required. These models should fit the electrophysiological data (Grassia et al., 2011). To interface in real-time biological assemblies and Spiking Neural Network (SNN), a real-time hardware implementation is therefore needed (Mahowald and Douglas, 1991; Indiveri et al., 2001; Levi et al., 2008). A biomimetic SNN is a neuromorphic system composed of high detailed level of analogy to the nervous system. It is based on biophysically detailed neurons, synapses, plasticity and noise. Most of them are in silicon (Ambroise et al., 2013) but some are also made with microfluidic techniques (Levi and Fujii, 2016). Biomimetic SNN is then one way to explore for designing new generation of neuroprosthesis and will facilitate the bio-hybrid experiments. Such components make one reconsider about the interaction between artificial devices and living cells.

With the appearance of real-time neuromorphic platforms, the desire to connect artificial neural networks with biological neural networks has emerged (Le Masson et al., 2002; Broccard et al., 2017). These systems often considers complex neuron models and plasticity and are in biological real-time. They mimic the action potential shape and the spike timing. Potter et al. (2014) and Levi et al. (2018c) presented different works on the closed-loop hybrid experiment. Potter et al. (2014) shows the latest innovations in the field, such as closed loop hybrid experiments using MEAs (Bareket-Keren and Hanein, 2012; Robinson et al., 2013), *in vitro* experiments (Bonifazi et al., 2013; Pimashkin et al., 2013), *in vivo* experiments (Opris et al., 2012; Nishimura et al., 2013) and clinical trials (Walter et al., 2012; Fernandez-Vargas et al., 2013). Vassanelli and Mahmud (2016) introduced the term “neurobiohybrid.” This term defines one system composed by at least two heterogeneous elements which one of them is biological neuron cells. These elements should communicate in a uni- or bidirectional way. Recently (Chou et al., 2014; Potter et al., 2014; Joucla et al., 2016; Serb et al., 2017) described different works on neurobiohybrid systems. This kind of interface would improve biomedical research and brain affliction (Bonifazi et al., 2013; Shahdoost et al., 2014; Capogrosso et al., 2016).

Understanding the behavior of neurons and their electrical activities, also called Action Potential (AP), is a key to design biomimetic systems. The choice of neuron model is the first difficult decision because it depends on the application of the systems and experiments.

The properties of the nerve cells can be mathematically described allowing the prediction of biological processes into a model called Spiking neuron model. Models of neurons that considers the spatial variation and the membrane potential of a neuron using several variables are called multi-compartmental. On the other hand, single compartmental models reproduces complex dynamics of real neurons including the evolution of ionic channels representing by conductances that is well-described by Hodgkin and Huxley (1952) using a four-dimensional set of equations. Such models require high computation resources generating suggestion of simplified models. Izhikevich (2003), Brette and Gerstner (2005), and Kohno et al. (2016) introduced two-dimensional models describing dynamical behavior of specific activities of neurons using simple equations.

Increasing the complexity and the dimension of the equations improves the biological plausibility; however, it increases the required resource and computation time. Bio-hybrid experiments require having an accurate/bio-realistic model which is able to reproduce the shapes and the frequencies of APs in Real-Time (RT). Biophysical experimental data (Alle and Geiger, 2006; Pospischil et al., 2008) suggest the possibility that variety of spikes given to a synapse plays a certain role in the information processing in the brain. For instance, Debanne et al. (2013) shows that the spike transmission and the modulation of neurotransmitter release are also a consequence of subthreshold presynaptic potential variations. Study of neurological disorders requires a bio-detailed model allowing one to modify biological parameters like reversal potentials, neuron size or ion conductance. Bio-detailed and bio-realistic are two different terms with different meanings. One represents the action potential according to biological and physical parameters and the other acts in the same way as the biology does. Adding synaptic noise (Destexhe et al., 2001; Grassia et al., 2016) and synaptic plasticity lead the model to a more accurate representation of a biomimetic neural network. Indeed, the noise generates stochastic behavior on the level of the neuronal dynamics, and influences the transmission of synaptic signals (Manwani and Koch, 1999) and impacts on the integrative properties of neurons (Stein et al., 2005).

SNN can be simulated with simulation software (Hines and Carnevale, 2001; Gewaltig and Diesmann, 2007; Goodman and Brette, 2009) and neuromorphic hardware. Power consumption and simulation time required to seek the solution are becoming relevant as neuroscientists turn to supercomputers to simulate brain-scale neural networks at cellular resolution. Today's supercomputers require 5 min to simulate 1 s of biological time on JUQUEEN (Kunkel et al., 2014; Jordan et al., 2018) which consumes 60–70 kW of power per racks, with 28 racks^1^, and 40 min for 1 sec on K^2^. In contrast, hardware implementations can realize real-time simulations with low power consumption. For bio-hybrid experiments, the choice of hardware systems is therefore more relevant.

Hardware implementations of SNN are divided into two major categories: mixed implementation (based on analog full-custom integrated circuits) and only digital implementation (based on FPGA, microprocessors, microcontrollers or neurochips). Moreover, the SNN could be (i) biomimetic, meaning it reproduces and imitates biological neurons, or (ii) bio-inspired meaning it is dedicated to computation tasks based on neural networks. In this paper, the system is based on biomimetic and non-bio-inspired systems.

In the case of mixed implementations, different neuron models are implemented: multi-compartmental (Hasler et al., 2007; George et al., 2013), conductance-based (Sorensen et al., 2004; Binczak et al., 2006; Renaud et al., 2007; Levi et al., 2018a; Natarajan and Hasler, 2018) or with threshold models (Liu and Douglas, 2004; Vogelstein et al., 2004; Indiveri and Fusi, 2007; Schemmel et al., 2007; Qiao et al., 2015; Kohno et al., 2016). Most of these systems are composed with an analog core for the neuron model implementation. The synapses and plasticity are usually integrated by digital techniques and the different analog cores are linked.

On the digital side, engineers and researchers are usually designing SNN for bio-inspired applications (Rice et al., 2009; Sabarad et al., 2012; Wang et al., 2013; Nanami and Kohno, 2016; Levi et al., 2018b). The number of implementations on the FPGA platform has been steadily increasing since 1997. Nazari et al. (2015) present the work of Cassidy et al. (2011) for an implementation of one million simple neurons, (Arthur et al., 2012) for the implementation of 256 Integrate-and-Fire neurons and 1,024 × 256 synapses, (Wang et al., 2013) for the implementation of 4,000 neurons and 1.15 million synapses.

Depending on the applications and on the choice of the neuron model, one main concern is the size of the neural network. Biological details of the neuron model are one constraint on the maximum number of neurons in the hardware system. To obtain medium size network of neurons (populations of around 1,000 neurons) or large-scale neural network (more than 10 000), it is necessary to:

- Implement a simple neuron and synapse models. Such implementation is presented by Cassidy et al. (2011) which shows a FPGA implementation of one million of neurons using the Leaky Integrate-and Fire model (LIF). Based on this research results, IBM has included one million neurons and 256 million synapses in his TrueNorth chip (Merolla et al., 2014).
- Perform neural network calculations, such as the SpiNNaker platform (Furber et al., 2013; Van Albada et al., 2018) with a multiprocessor architecture and real-time computations using an integration step of 1.0, 0.1 ms is classic for neuroscience applications. Another system called BrainScaleS perform calculations of neural network 104 times faster than biological time (Rast et al., 2013). BrainScaleS is composed of several modules including wafer with 448 neuromorphic chips and a routing system achieving a simulation of 512 neurons activities and 115,000 synapses. Both systems are used for the Human Brain Project (Markram, 2012). They performed high-speed simulations of the brain but no bio-hybrid experiments.

The Hodgkin-Huxley (HH) equations have been the subject of studies for FPGA implementation. According to the computation methodology used for the design, the computation time, the resources and the precision can be modified. Osorio (2016) present a pipelined implementation using floating point numbers and complex methods, such as the Runge-Kutta algorithm to solve the differential equation, Taylor series for the exponential and Goldsmith algorithm. However, such methodology requires a lot of FPGA resources. Bonabi et al. (2014) showed good precision using fast and low resources algorithms, such as the CORDIC algorithm and Euler method for the computation of 120 neurons connected by synapses. More recently, Akbarzadeh-Sherbaf et al. (2018) presented another way to compute HH neurons on FPGA with 5,120 real-time neurons (or 65,536 using a different time scale).

Such complex models need an optimization and an adaptation to the digital implementation. It is important, according to the application, to know how far the approximations and the modifications could modify the behavior of the SNN. Several different tools exist that allows us to compare biological data with the device output, such as bifurcation analysis, cross-correlation, and frequency to stimulation or amplitude to stimulation graph. Here, we present the first optimized implementation of the HH formalism adapted to different classes of cortical neurons on FPGA including synaptic noise and synapses. We also propose a low resource and high-speed digital architecture allowing biomimetic features in real time.

## Materials and Methods

Different types of cortical neurons have been identified (Izhikevich, 2003) and described (Gutnick and Mody, 2012) in term of AP shape and frequency. Among them the Fast Spiking neuron (FS), Regular Spiking neuron (RS), Intrinsically Bursting neuron (IB), and Low-Threshold Spiking neuron (LTS) are the main focus and they are described in this paper. The model used in this paper is based on a simple-compartment model of HH with the parameters of Pospischil et al. (2008). Here, we proposed a SNN based on these equations and adapted to digital hardware allowing the computation of cortical neurons connected by synapses including synaptic noise to incorporate spontaneous activities. Also, different tools have been developed to validate our system and to compare it with biological recordings. The second part will show how we validated the system, how errors can be calculated and at which moment the mistake from the optimization can be acceptable and how close to the biology it should be and can be.

### Neural Network Model

#### Neuron Model

**Figure 1:**
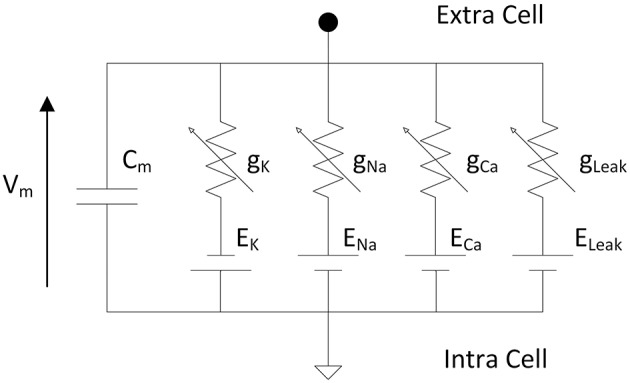
Electrical circuit representing the membrane of one neuron according to Hodgkin and Huxley where Vm is the membrane voltage, Cm the membrane capacitance, g_K_, g_Na_, g_Ca_ are the variable conductances representing the opening/closing gate of the ionic channels and E_K_, E_Na_, E_Ca_ are the reversal potentials of the different ions. The leakage conductance composed of g_Leak_ and E_Leak_ maintains the resting potential of the neuron.

Hodgkin and Huxley proposed an equivalent circuit (Figure 1) of a nerve membrane which can reproduces action potential and dynamics of ionic channels (Hodgkin and Huxley, 1952). The model described differential equations from an electrical circuit representing the membrane of one neuron. The membrane voltage is a function of the membrane capacitance and the ionic currents.

The HH formalism is described here using the parameters of Pospischil et al. (2008). It reproduces the biological behavior of different cortical neurons: FS, RS, IB, and LTS neurons. The equations include several ionic currents to simulate other dynamic effects than the original model and to reproduce the complex dynamic behaviors of cortical neurons.

The Equations (1–6) describe five ionic currents corresponding, respectively to potassium (I_K_), sodium (I_Na_), leakage (I_Leak_), slow potassium (I_Kp_) for frequency adaptation and calcium (I_CaL_ for I_B_ or I_CaT_ for LTS).

(1) $$\begin{array}{l} C_{m}\frac{dV_{m}}{dt}=I_{s}-\underset{i}{\sum }I_{i} \end{array}$$

where V_m_ is the membrane voltage, C_m_ the membrane capacitance, I_s_ the stimulation current in μA/cm^2^ and I_i_ (I_Na_, I_K_, I_Kp_, I_CaL_) the ionic current that can be represented by (2). I_CaT_ is defined by (3).

(2) $$\begin{array}{l} I_{i}=\bar{g_{i}}\times m^{p}\times h^{q}\times \left(V_{m}-E_{i}\right) \end{array}$$

(3) $$\begin{array}{l} I_{CaT}=\bar{g_{CaT}}\times {m_{\infty }}^{\text{2}}\times h\times \left(V_{m}-E_{Ca}\right) \end{array}$$

(4) $$\begin{array}{l} \frac{dx}{dt}=\frac{x_{\infty }\left(V_{m}\right)-x}{\tau_{x}\left(V_{m}\right)} \end{array}$$

where x ∈ {m, h}, τ_*x*_(*V*_*m*_) and *x*_∞_(*V*_*m*_) are obtained by the Equations (5, 6), p and q are p = 4, q = 0 for I_K_, p = 3, q = 1 for I_Na_, p = 1, q = 0 for I_M_ and p = 2, q = 1 for I_CaL_. The variable m is the activation and h the inactivation probability of the voltage dependent channel.

(5) $$\begin{array}{l} \tau_{x}\left(V_{m}\right)=\frac{1}{\alpha_{x}\left(V_{m}\right)+\beta \left(V_{m}\right)} \end{array}$$

(6) $$\begin{array}{l} x_{\infty }\left(V_{m}\right)=\frac{\alpha_{x}\left(V_{m}\right)}{\alpha_{x}\left(V_{m}\right)+\beta \left(V_{m}\right)} \end{array}$$

(6) where α and β are exponential based equations. α and β parameters are given in Pospischil et al. (2008).

The parameters used in the above equations are shown on Table 1 and are described in Pospischil et al. (2008). These equations have been implemented on Matlab and adapted from the implementation of Alain Destexhe on the Neuron software.

**Table 1:** Values used for computation of the HH model using Pospischil parameter.

	**FS**	**RS**	**IB**	**LTS**
dneur (μm)	67	96	96	96
Sm (cm^2^)	π × *dneur*^2^ × 10^−8^			
Cm (μF/cm^2^)	1			
Is (μA/cm^2^)	3.54	2.59	0.51	1.24
*g_k_* (mS/cm^2^)	10	5	5	5
*g*_*Na*_ (mS/cm^2^)	50	50	50	50
*g*_*Leak*_ (mS/cm^2^)	0.15	0.1	0.01	0.01
*g*_*M*_ (mS/cm^2^)	0	0.07	0.03	0.03
*g*_*CaL*_ (mS/cm^2^)	0	0	0.17	0
*g*_*CaT*_ (mS/cm^2^)	0	0	0	0.4
*E*_*k*_ (mV)	−100	−100	−100	−100
*E*_*Na*_ (mV)	50	50	50	50
*E*_*Leak*_ (mV)	−70	−70	−85	−50
*E*_*Ca*_ (mV)	0	0	120	120
V_Init_ (mV)	−70	−70	−84	−84

Considering the neuron as a cylinder, dneur and Sm are the size of neuron; I_s_ has been calculated using the current in μA divided by Sm; V_init_ represents the value of the first point.

#### Synapses

Neurons communicate using Action Potential through small units called synapses that interface with axons and dendrites. Reproducing a biomimetic neural network implies the study and the realization of the synapses' behavior. Binding chemical messages, the synapse receptors AMPA, GABAa, GABAb and NMDA are responsible for synaptic transmissions and their activities has been recorded and modeled (Destexhe et al., 1998). The following equations represent the synaptic current of excitatory synapses (AMPA and NMDA) and inhibitory synapses (GABA_a_ and GABA_b_).

(7) $$\begin{array}{l} I_{AMPA}={\bar{g}}_{AMPA}.r.\left(V_{post}-E_{AMPA}\right) \end{array}$$

(8) $$\begin{array}{l} I_{NMDA}={\bar{g}}_{NMDA}.r.B\left(V_{pre}\right).\left(V_{post}-E_{AMPA}\right) \end{array}$$

(9) $$\begin{array}{l} I_{GABAa}={\bar{g}}_{GABAa}.r.\left(V_{post}-E_{GABAa}\right) \end{array}$$

where,

(10) $$\begin{array}{l} \frac{dr}{dt}=\alpha .\left[T\left(V_{pre}\right)\right].\left(1-r\right)-\beta .r \end{array}$$

(11) $$\begin{array}{l} I_{GABAb}={\bar{g}}_{GABAb}.\frac{s^{n}}{s^{n}+K_{d}}.\left(V_{post}-E\right) \end{array}$$

where,

(12) $$\begin{array}{l} \frac{ds}{dt}=K_{3}.r-K_{4}.s \end{array}$$

(13) $$\begin{array}{l} \frac{dr}{dt}=K_{1}.\left[T\left(V_{pre}\right)\right].\left(1-r\right)-K_{2}.r \end{array}$$

I_x_ is the synaptic current with x representing the AMPA, NMDA, GABA_a_, or GABA_b_ neurotransmitter. V_post_ is the membrane voltage from the post-synaptic neuron received by the membrane voltage V_pre_ from the presynaptic neuron sending the action potential. α, β, Kd, K1, K2, K3, K4, and n are constants and g_x_, with x representing the neurotransmitters, is the conductance. All the equations and the parameters can be found on (Destexhe et al., 1998). The computation of T can be optimized using the method shown in section Optimization Toward Implementation by a hyperbolic tangent. The parameters are α = 1.1, β = 0.19, g = 0.9 nS, E = 0 mV for AMPA, α = 0.072, β = 0.00066, g = 0.35 nS, E = 0 mV for NMDA, α = 5, β = 0.18, g = 1 nS, E = −70 mV for GABAa and K1 = 0.09, K2 = 0.0012, K3 = 0.18, K4 = 0.034, Kd = 100, n = 4, g = 1 nS and E = −95 mV.

The different receptors show various behavior that can be found in the nerve system. AMPA and NMDA are excitatory synapses which respectively induce fast and slow excitation. On the other hand, GABAa and GABAb are inhibitory synapses which respectively produce fast and slow inhibition. These effects can be observed on the FPGA in the result section of this article and in **Figure 11**.

#### Noise

*In vivo* experiments show that neurons have noise behaviors both in the transmission of synaptic signals and in the generation of action potentials. Subthreshold membrane oscillations can be observed. This noise behaviors come from intrinsic or extrinsic sources (Manwani and Koch, 1999) or from the thermal noise. Commonly, computational neuroscientists modeled synaptic activity using fluctuating conductance (Destexhe et al., 2001), or by adding a source of noise current in the neuron model (Levitan et al., 1968; Tuckwell, 1988). Hence, the neuron activity can be modeled by stochastic differential equations. In previous work (Grassia et al., 2016), a FPGA implementation of quartic model was suggested using Ornstein–Uhlenbeck process as source of current noise. For this work, we will use the same process adapting the parameters for the Hodgkin-Huxley model. The Ornstein–Uhlenbeck process is described by the following differential equation:

(14) $$\begin{array}{l} dI\left(t\right)=\theta \cdot \text{Δ}t\cdot \left(\mu -I\left(t\right)\right)+\sigma \cdot dW\left(t\right) \end{array}$$

Where, Wt denotes the Wiener process, θ > 0, σ > 0 are parameters and μ represents the mean value or the equilibrium for the process.

The form (15) represents the stationary variance of Ornstein–Uhlenbeck process:

(15) $$\begin{array}{l} \mathrm{var}\left(X_{t}\right)=\frac{\sigma^{2}}{2\theta } \end{array}$$

(15) The source of current noise described in the stochastic differential Equation (14) can be considered as an approximation of the results from opening and closing of the channel that is controlled by a set of independent gating particles on a neuron's surface (Tuckwell et al., 2002). Using Euler–Maruyama method (Higham, 2001), an explicit integration algorithm for FPGA implementation (Grassia et al., 2016) was obtained. Consequently, the approximation to the solution can be recursively described for 1 ≤ n ≤ N as in this way:

(16) $$\begin{array}{l} \text{I}\left[\text{n}+1\right]=\text{I}\left[\text{n}\right]+\text{θ}\left(\text{μ}-\text{I}\left[\text{n}\right]\right)\text{Δt}+\text{σΔW}\left[\text{n}\right] \end{array}$$

where, ΔW[n] are independent and identically distributed random variables with expected value zero and variance Δt, thus ΔW[n]~N(0,Δt) = √Δt^*^N(0,1); Δt = T/N is the time step after the partition of the interval [0, T] into N equal subintervals of width Δt > 0, n is the iteration step. To realize an FPGA implementation of the Euler-Maruyama method, normally distributed random variables with standard deviation √Δt were generated.

The Equations (14, 16) are represented under the form of a current to add with the ionic current. In the result section, the neuron dynamic with synaptic noise will be discussed.

## Optimization Toward Implementation

Unlike low-level implementation, computation of ionic channel is costly due to the presence of extensive calculations of exponentials and divisions. Here, optimized equations for the ionic dynamics adapted to a low-level digital implementation that are low cost are introduced. Optimizing the equations reduces the computation time and increases the number of neurons, synapses and other features like the noise in the FPGA.

### Alternative Equations

Another method of representing the equations is by fitting *x*_∞_ and τ_*x*_ using hyperbolic functions. This choice of using hyperbolic functions comes from the development of a high speed and low resources module on FPGA for reproducing hyperbolic functions using CORDIC algorithm (see part FPGA Implementation). Thus, ionic channels can be represented by the same functions with different parameters.

(17) $$\begin{array}{l} \frac{dx}{dt}=\frac{x_{\infty }\left(V_{m}\right)-x}{\tau_{x}\left(V_{m}\right)} \end{array}$$

(18) $$\begin{array}{l} x_{\infty }\left(V_{m}\right)=a_{1}.\tanh\left(b_{1}.\left(V_{m}-V_{x}\right)+c_{1}\right)+d_{1} \end{array}$$

(19) $$\begin{array}{l} \tau_{x}\left(V_{m}\right)=\frac{a_{2}}{\cosh\left(b_{2}\times \left(V_{m}-V_{x}\right)+c_{2}\right)}+d_{2} \end{array}$$

Where x is the concerned ionic channel (m or h), V_m_ the membrane voltage and a, b, c, d are parameters. The equations present different constants, which are represented on Tables 2, 3 according to the ionic channel and the ionic current needed.

**Table 2:** Parameters for **x**_∞_; The computation of an IB and a LTS neuron requires more precision on *m*_∞_, then the parameters become: a_1_ = 0.001179^*^v + 0.5479, b_1_ = 2^−4^ + 2^−10^, c1 = 1.954 and d1 = 0.4577 when Vmem < −30 else, a_1_ = 0.0001474^*^v + 0.4383, b_1_ = 2^−4^ + 2^−8^, c1 = 1.817, and d1 = 0.567.

		***a*_1_**	***b*_1_**	***c*_1_**	***d*_1_**
I_K_	m	2^−1^	2^−5^ + 2^−6^	−1.128	0.467
I_Na_	m		2^−4^	−1.718	0.497
	h		−2^−3^	2.705	0.502
I_M_	m		2^−5^ + 2^−6^	−0.938	0.5
I_L_	m		2^−3^	4.151	0.495
	h		−2^−6^ – 2^−7^	2.705	0.502

**Table 3:** Parameters for τ_x where γ = −0.2334 × (V_m_ – V_x_) + 9.649, δ = −0.1118 × V_m_ + 2.264 and ε = −0.9446 × V_m_ + 237.6; Vx = 2 mV.

		***a*_2_**	***b*_2_**	***c*_2_**	***d*_2_**
I_K_	*m*	1.325	0.046	−0.497	0.335
I_Na_	*m*	0.893	−0.041	0.937	0.029
	*h*	γ	0.099	−1.960	0.3498
I*_*M*_*	*m*	2^−1^	−1.618	2^−4^	0.498
I_CaL_	*m*	δ	0.093	3.452	0.371
	*h*	ε	−0.029	−1.833	150.7

The ionic current ICaT follows the same rules. The differences come from the fact that τ can be calculated by another hyperbolic tangent. Thus, *m*_∞_, *u*_∞_ and τ_*u*_ can be calculated with the following formula using the parameter in Table 4.

(20) $$\begin{array}{l} f\left(v\right)=a.\tanh\left(b.v+c\right)+d \end{array}$$

**Table 4:** Parameters for the computation of I_CaT_ ionic current.

	**a**	**b**	**c**	**D**
*m*_∞_	2^−1^	2^−4^ + 2^−6^	5	0.5
*u*_∞_		−2^−3^	−11	
τ_*u*_	−28.09	0.162	12.808	37.03

**Figure 2:**
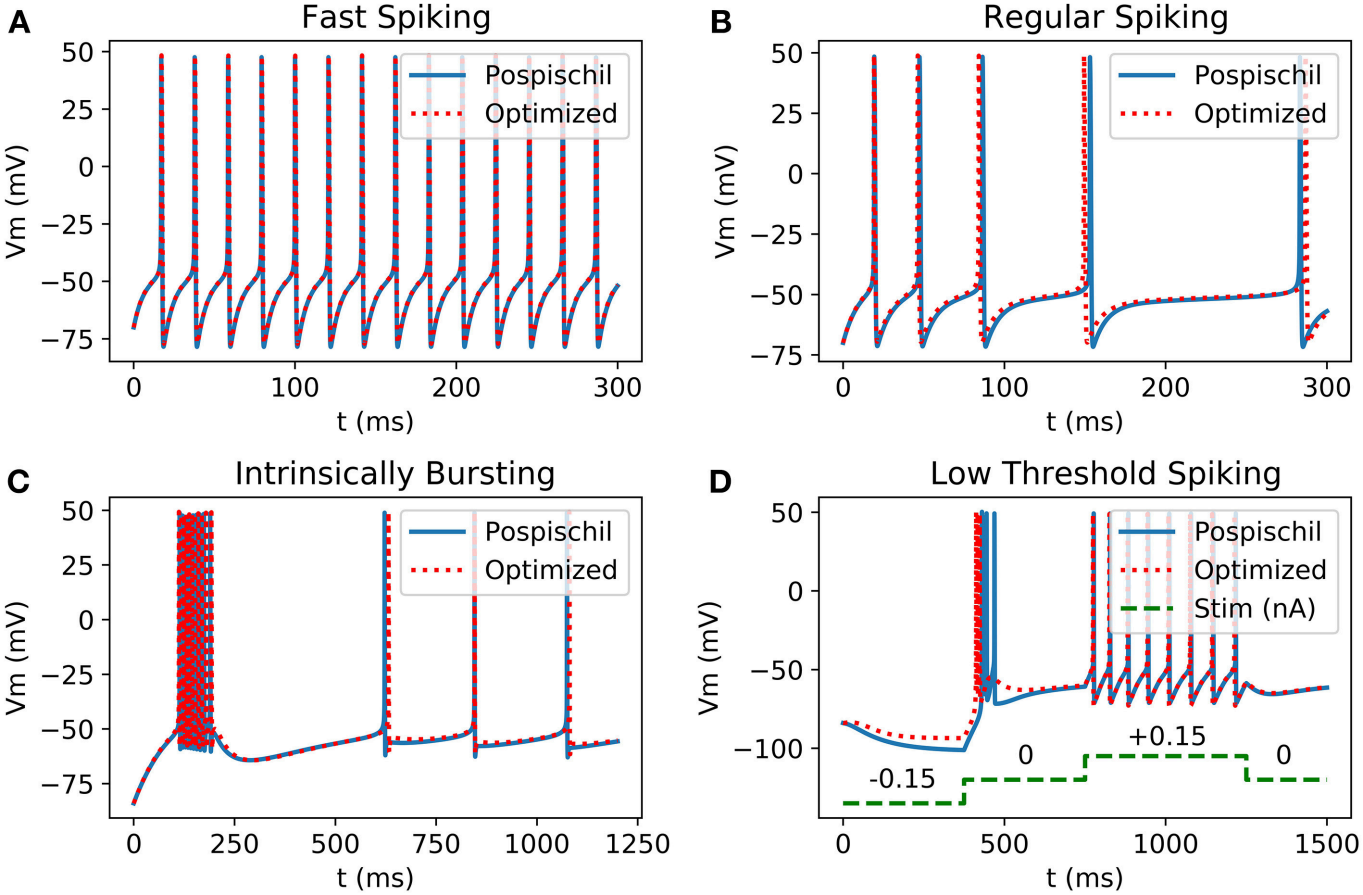
Comparison between Matlab implementation of HH neurons using Pospischil parameters and Matlab implementation of simplified model: **(A)** Fast Spiking neuron possesses a constant frequency while the current stimulation is constant; **(B)** Regular Spiking neuron shows a frequency adaptation; **(C)** Intrinsically bursting neuron begins with a burst when the threshold is exceeded; **(D)** Low Threshold Spiking burst after when a negative excitation ends and has a regular behavior when excited with positive current, the burst of the optimized result shows less spikes than the original version.

These novel equations and calculation methods are able to reproduce action potential (Figure 2) using unifying equations and present small differences in frequency and amplitude. Using the power of two as coefficient allows the replacement of multiplier by logical shift operations which makes the implementation lighter and faster. Also, hyperbolic tangent and cosine are faster to compute than exponentials.

In order to have similar amplitude and frequency for all the classes of neurons, best stimulation current minimizing the root mean square error and maximizing the R value of cross-correlation has been found.

(21) $$\begin{array}{l} f_{original\text{\_}FS}\left(Is\right)=f_{Simplified\text{\_}FS}\left(Is\right)=\;\left(7.58.Is\right)+3.381 \end{array}$$

(22) $$\begin{array}{l} {\;f}_{original\text{\_}RS}\left(Is\right)=\left(4.3.Is\right)-0.66\text{;}\\ f_{Simplified\text{\_}RS}\left(Is\right)=\left(4.206.Is\right)-0.661 \end{array}$$

where, I_s_ is the stimulation current.

The optimized version of Hodgkin-Huxley model shows similar behavior in shape and in frequency as the original one's. Modifying the Hodgkin-Huxley equation requires one to measure the correlation between the two signals and the stability of the new system compared to that of the original one.

### Validation Tools

The proposed set of equations are chosen and validated to be close to the original equations and to the biological behavior. It is modeled to be convenient in term of resources and time computation as a digital implementation. Different ways of comparison have been developed for showing similarities between the dynamics of two set of data. Frequency vs. Stimulation current curve (F-I) and Amplitude vs. Stimulation current curve (A-I) are the most representative because they show the different stimulation currents of reaction of neurons. Moreover, it is possible to compare the dynamics of the living cells to the artificial cells. The F-I graph shows strong similarities for the FS between the biological recording and the FPGA output which represents the frequency of the neuron according to the stimulation.

**Figure 3:**
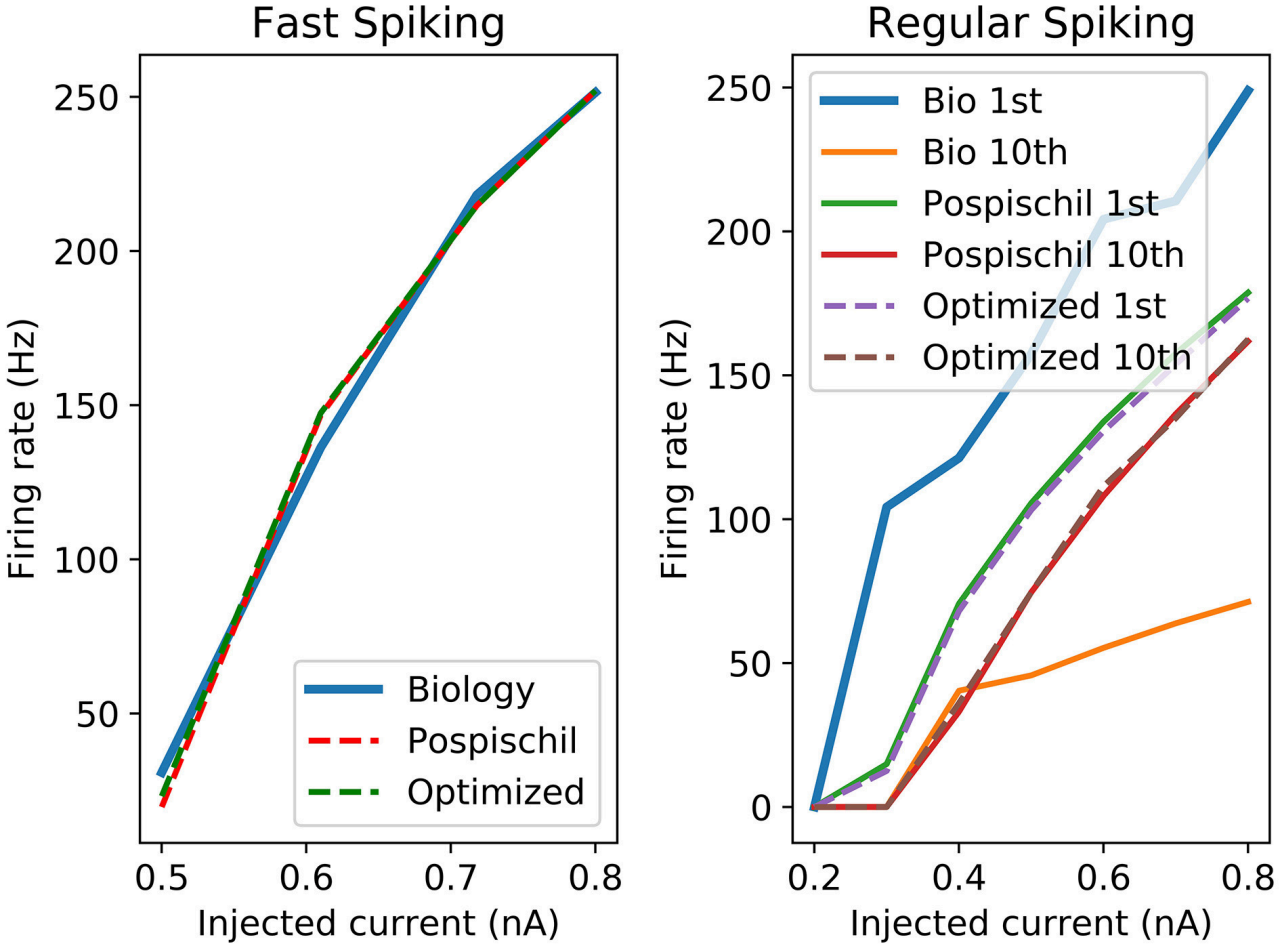
Left graph shows the F-I curve of the biological cell recordings (Blue curve named Biology), original model (red dotted line named Pospischil) from Pospischil et al. (2008) and the optimized model proposed in this paper (green dotted in named Optimized); Right graph shows the frequency of the first and 10th spike of the original and optimized equations compared to the biological cell.

F-I curves (Figure 3) show good correlation between the original and the optimized equations. However, as Pospischil et al. (2008) stated, the model does not fit with the biological recordings but shows frequency adaptation which is used for the other type of neurons. F-I shows the behavior of an isolated neuron when applying a stimulation current. However, it does not consider the shape of the AP. Optimized neuron implementation will show similar behavior to the model which aims to fit electrophysiological recording. However, how similar is the optimized implementation from reality and how far from the reality should the AP be? These common questions about correlation have been the object of studies and show that one of the known tools is the Pearson Product Moment Correlation (PMCC) (Cohen and Kohn, 2013). Pearson correlation gives a value between −1 (negative correlation) and +1 called “r.” Other tools have been used to analyze brain signals going from the electrophysiology records to the neuroimaging results (Ide et al., 2017). Statistical method, such as Granger causality analysis can be used to predict one series from other series (Seth et al., 2015) despite a delay in signals. Also, cross-correlation (CC) is one of the most common correlation methods used in different fields which quantifies the correlation and the phase difference of two signals, also called lag (Yuan et al., 2015). Pearson correlation is defined by the Equation (3) and shows the covariance divided by the multiplication of the standard deviation of the two set of data called x and y.

(23) $$\begin{array}{l} r_{xy}=\frac{1}{N-1}\overset{N}{\underset{i=1}{\sum }}\left(\frac{\bar{x_{i}-\mu_{x}}}{\sigma_{x}}\right)\left(\frac{y_{i}-\mu_{y}}{\sigma_{y}}\right)\; \end{array}$$

where μ_*x*_ and μ_*y*_are respectively the mean of x and y, σ_*x*_, and σ_*y*_ are the standard deviation of x and y, N the number of samples.

Cross-correlation is similar to the Pearson correlation but adds the lag as a feature. The function is also close to a convolution product that translates a signal and measures its similarity with a reference signal.

(24) $$\begin{array}{l} r_{xy}\left(i\right)=\overset{N}{\underset{j=0}{\sum }}x\left(i\right).y\left(i+j\right) \end{array}$$

where N the number of points, i and j are the index of the data vectors.

**Figure 4:**
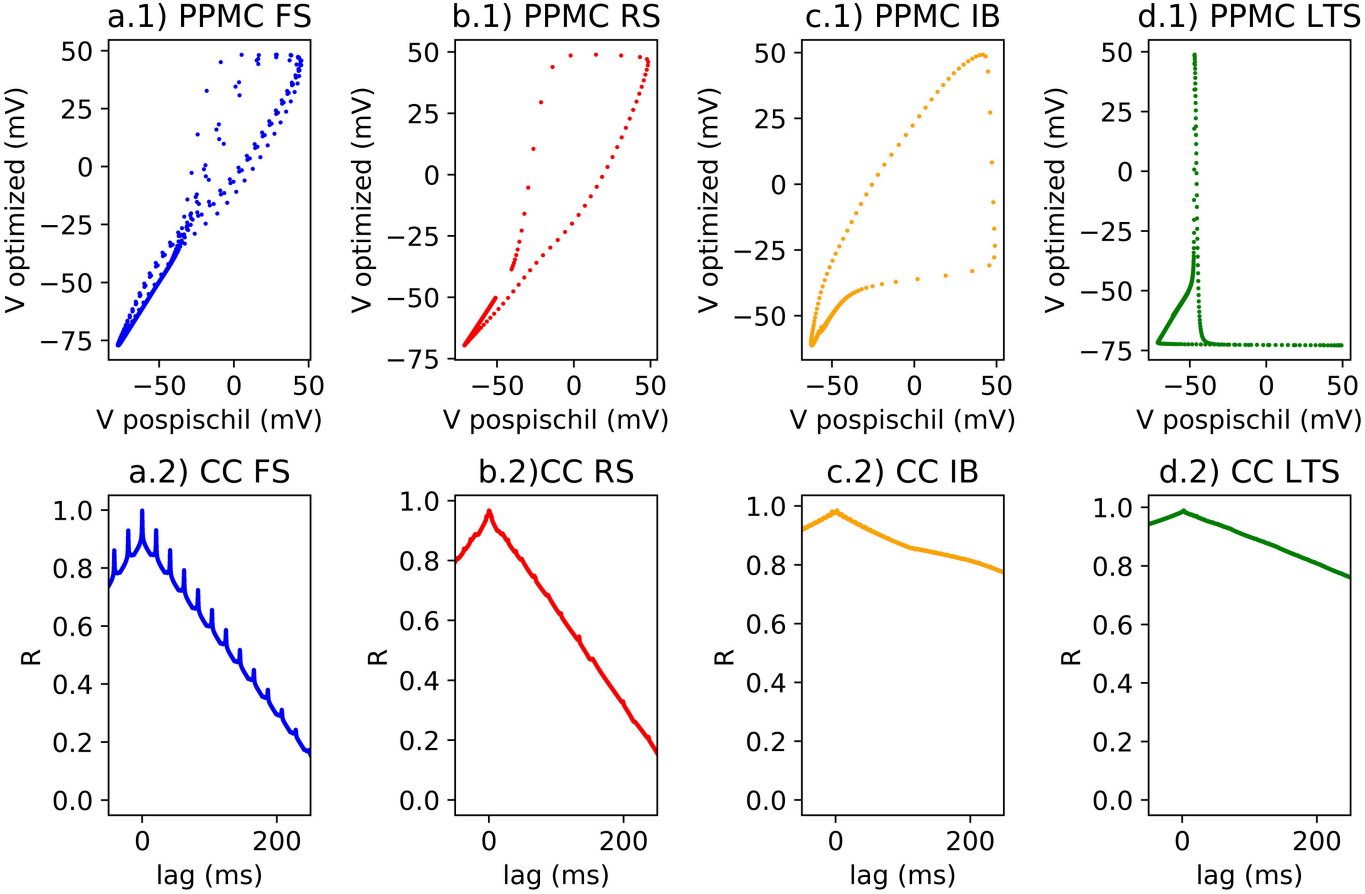
(1) Single spike on scatter plot for the Pearson Correlation calcul; (2) Lag and R coefficient represented for each classes of neurons; **(a)** FS neuron with a stimulation of 0.5 nA, r_PPMC_ = 0.96 for a single spike and for a spike train, r_CC_ = 0.99; **(b)** RS neuron with a stimulation of 0.75 nA, r_PPMC_ = 0.96 for a single spike, r_PPMC_ = 0.37 for a spike train, r_CC_ = 0.97; **(c)** IB neuron with a stimulation of 0.15 nA, r_PPMC_ = 0.89 for a single spike, r_PPMC_ = 0.34 for a burst, r_PPMC_ = 0.38 for a spike train, r_CC_ = 0.97; **(d)** LTS neuron with a stimulation of −0.15 nA, then no stimulation, then 0.15 nA of stimulation, r_PPMC_ = 0.38 for a single spike, r_PPMC_ = 0.34 for a burst, r_PPMC_ = 0.38 for a spike train, r_CC_ = 0.97.

Cross-correlation is simple and sufficient for small size of the neural network capable of quantifying the differences between original equations computed on MATLAB and ones computed on FPGA (Figure 4).

Pearson correlation identifies the correlation >0.9 compared to a single spike of each classes of neuron. However, when applying train of spike, the r value decreases (Figures 4a.1–d.1). The error could be originated from a small phase induced by the modification of the model which is demonstrated by the (Figures 4a.2–d.2). The R value of the CC of two compared signals suggests better correlation (>0.9) with a |lag| different to 0.

The proposed set of equations for the Hodgkin-Huxley model are validated to be close to the original equations through bifurcation analysis that is used to investigate the dynamical property of the neuron model in response to a sustained current stimulus as shown by Izhikevich (2000) and by Rinzel and Ermentrout (1989). Biological neurons can be classified into Class 1 or Class 2 according to their response to a stimulation current (Hodgkin, 1948). The characteristic of Class 1 is observed when the transition from non-active phase to spiking phase is associated with a saddle-node bifurcation. When resting state loses its stability via a Hopf bifurcation, biological neurons classified as Class 2. The dynamical properties of the HH model that have been studied by Hassard (1978) and Hassard et al. (1981) belong to Class 2. As already investigated in previous work (Grassia et al., 2012), AUTO software was used to perform bifurcation analysis for equilibria as well as for periodic orbits, to compare our simplified HH model to that of Hansel et al. (1993).

**Figure 5:**
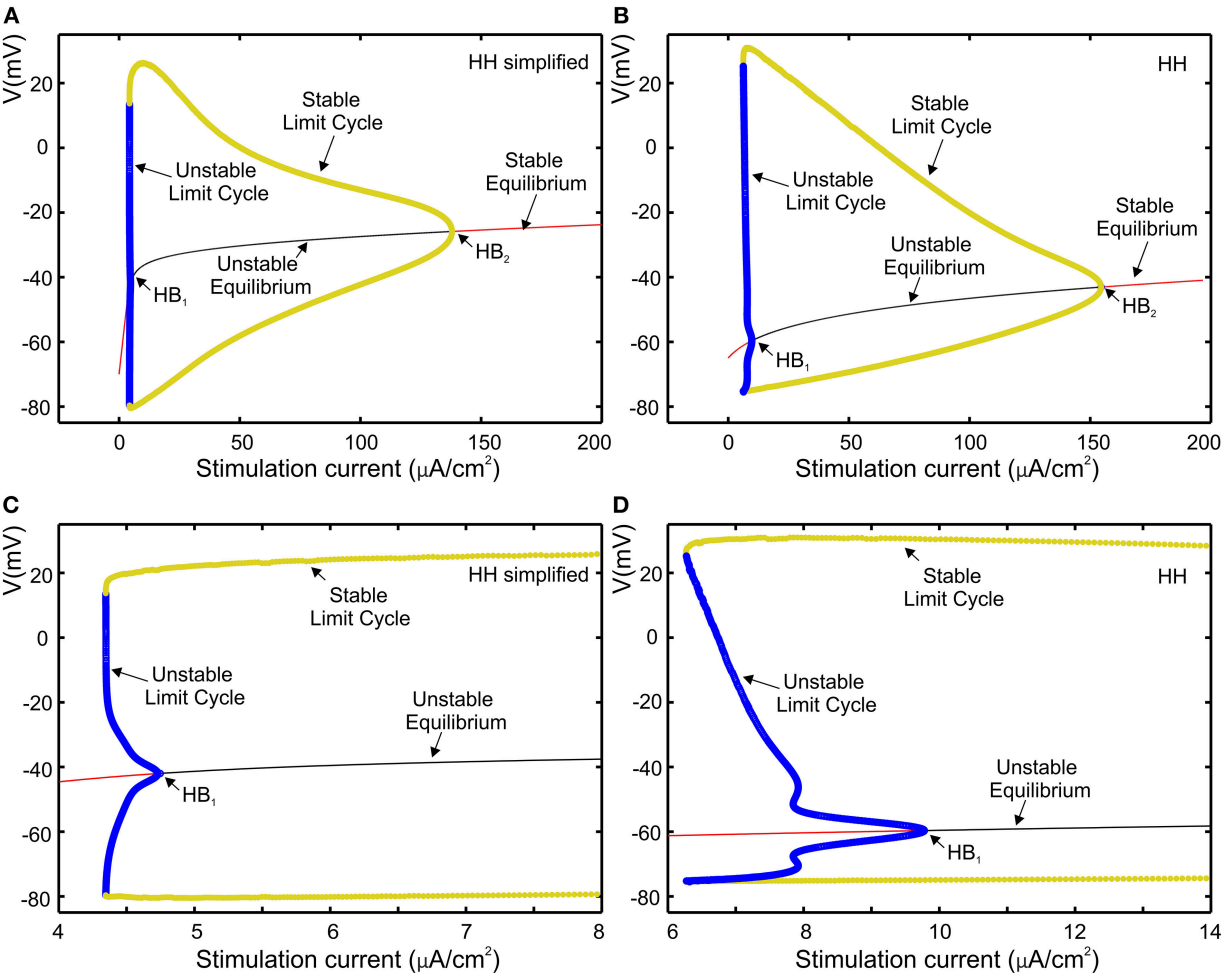
Comparison of the models using bifurcation analysis. **(A)** Bifurcation diagram of our simplified HH model. **(B)** Bifurcation diagram for HH model; **(C,D)**, subcritical Hopf bifurcation at the first HB point, respectively zoomed view of **(A,B)**.

Bifurcation diagram of our simplified HH model and the original HH model are shown respectively in Figures 5A,B, where red and black curves define stable and unstable equilibria, respectively, while yellow and blue curves respectively represent stable and unstable limit cycles. The repetitive firing that emerges when sustained stimulation current was applied corresponds to stable limit cycle. It suggests that in both cases the limit cycles begin initially through a fold bifurcation of limit cycles, then at the Hopf bifurcation (HB) point the systems state jumps to the stable limit cycle. Figures 5C,D represent a zoomed view of Figures 5A,B in which we can observe that, via the subcritical Hopf bifurcation, the resting state loses its stability. Furthermore, in both case, we observed that through a fold bifurcation a limit cycle a limit cycle appears and disappears by a supercritical Hopf bifurcation at the second HB point. Moreover, the reduction in amplitude is similar in both cases as shown in Figures 5A,B.

The bifurcation analysis affirms that our simplified HH model shares the dynamics of the HH model while keeping the non-linear dynamical characteristics of the original model.

## FPGA Implementation

What are the requirements for designing electrical system for bio-hybrid platforms and neurological diseases study. It must be must a tunable system allowing to easily and quickly simulate different kinds of neurons with different parameters. It must be a low resources system that allows additional parameters like synapses and noise. Finally, having a system containing a large number of neurons is useful for the study of simulation of brain regions, robotics application and different network dynamic behaviors. The FPGA is the best component due to its flexibility, speed and stability. Thus, the novel equations have been designed to be adapted to a FPGA implementation. To do so, basic operations, such as exponential, hyperbolic functions, division, differential equation resolution or multiplication must be designed using complex algorithms. Here, the hardware is used to output the AP in order to show the different algorithms and methods to compute these operations.

### System

The chosen FPGA is a Kintex-7 of “xc7k325tffg900” family included in the Genesys 2 evaluation board. In order to output the signals, a Digital to Analog Converter (DAC) with a precision of 12 bits and a 2.5 V reference voltage converts the digital data. The frequency of the system is 100 MHz.

Fixed point coding has been used for the calculations. It is basically composed by 1 bit of sign, 7 bits of integer and 10 bits of decimal for the membrane voltage. The size of the vectors is limited by the DSP of the Kintex-7 which computes the multiplication of an 18 bits by a 24 bits vector. According to the need of precision concerning the different parts of the system, the size should not be the same. For example, the calculation of the sodium conductance (m and h computation) has been designed with a 18 bits vector containing 1 bit of sign, 1 bit of integer and 16 bit of decimal. Ionic channels computation requires more precision and the values will never be up to 1 because its nature of probability of opening/closing of ionic gate.

### Mathematical Tools

The differential equations can be solved using the Euler method (25) which defines derivative function. Looking at the equations, it can be deduced that the smaller the dt is, the higher is the precision. The value of dt is chosen to be digitally convenient, to have stable dynamics and to stay in the acceptable range of living variability. The following equations show that the resolution of the differential equation needs one multiplier and one adder.

(25) $$\begin{array}{l} \text{f}\left(\text{t }+\text{ dt}\right)=\text{f}\left(\text{t}\right)+\text{ dt}.{\text{f}}^{\prime }\left(\text{t}\right) \end{array}$$

(26) $$\begin{array}{l} {\text{f}}_{\text{i}+1}={\text{f}}_{\text{i}}+\text{ dt}.{{\text{f}}_{\text{i}}}^{\prime } \end{array}$$

**Figure 6:**
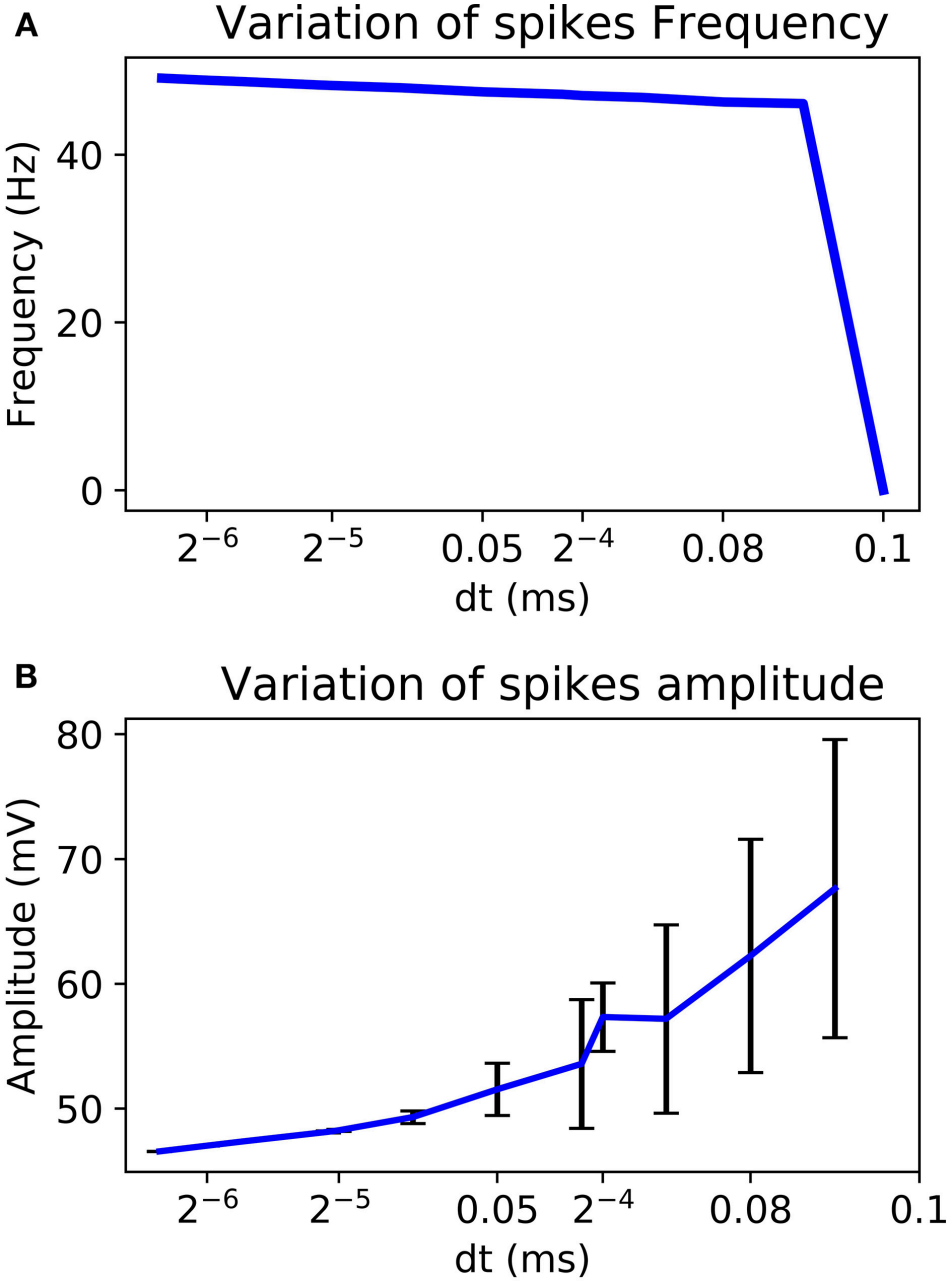
Variability of the frequency **(A)** and the amplitude **(B)** of AP according to the chosen dt for Euler method.

By choosing a power of two as dt it is possible to replace the multiplier by a logical shift operation. Also, according to the chosen dt, amplitude and frequency of the AP can change. Figure 6 shows a better stability with few variations for a smaller value of dt. It also analyses the variability of the system according to the chosen value.

Division has been the subject of much research and several algorithms have been developed in order to increase the performance of the operation on a computer (Obermann and Flynn, 1997). Among them, non-restoring division is a simple and efficient way to compute a division of fixed point number on a digital platform (Sutter and Deschamps, 2009).

Multiple algorithms exist to compute hyperbolic functions, such as COordinate Rotation DIgital Computer (CORDIC) which is mostly used in digital implementation. This method is optimized for low resources and high-speed computation (Andraka, 1998). CORDIC algorithms allow the calculation of hyperbolic sine and cosine from which it is possible to calculate the exponential and the hyperbolic tangent by coupling the Non-restoring division with the CORDIC module using the following equation:

(27) $$\begin{array}{l} e^{x}=\text{ cosh}(x)+\text{ sinh}\left(x\right) \end{array}$$

(28) $$\begin{array}{l} \text{tanh}\left(x\right)=\frac{\text{sinh}\left(x\right)}{\text{cosh}\left(x\right)} \end{array}$$

CORDIC and Non-Restoring algorithm are iterative methods. They need multiple stages before retrieving the result. When designing the FPGA module, using iteration would take more computation time or more resources. In order to limit both time and resources, the designed computation block for division and hyperbolic operations uses multiple stages of operations by performing in one clock cycle. Due to number of stages, it is better to avoid the use of these modules when it is possible to limit the number of iterations and to limit the number of bits of the vectors used in the operations.

### FPGA Architecture

**Figure 7:**
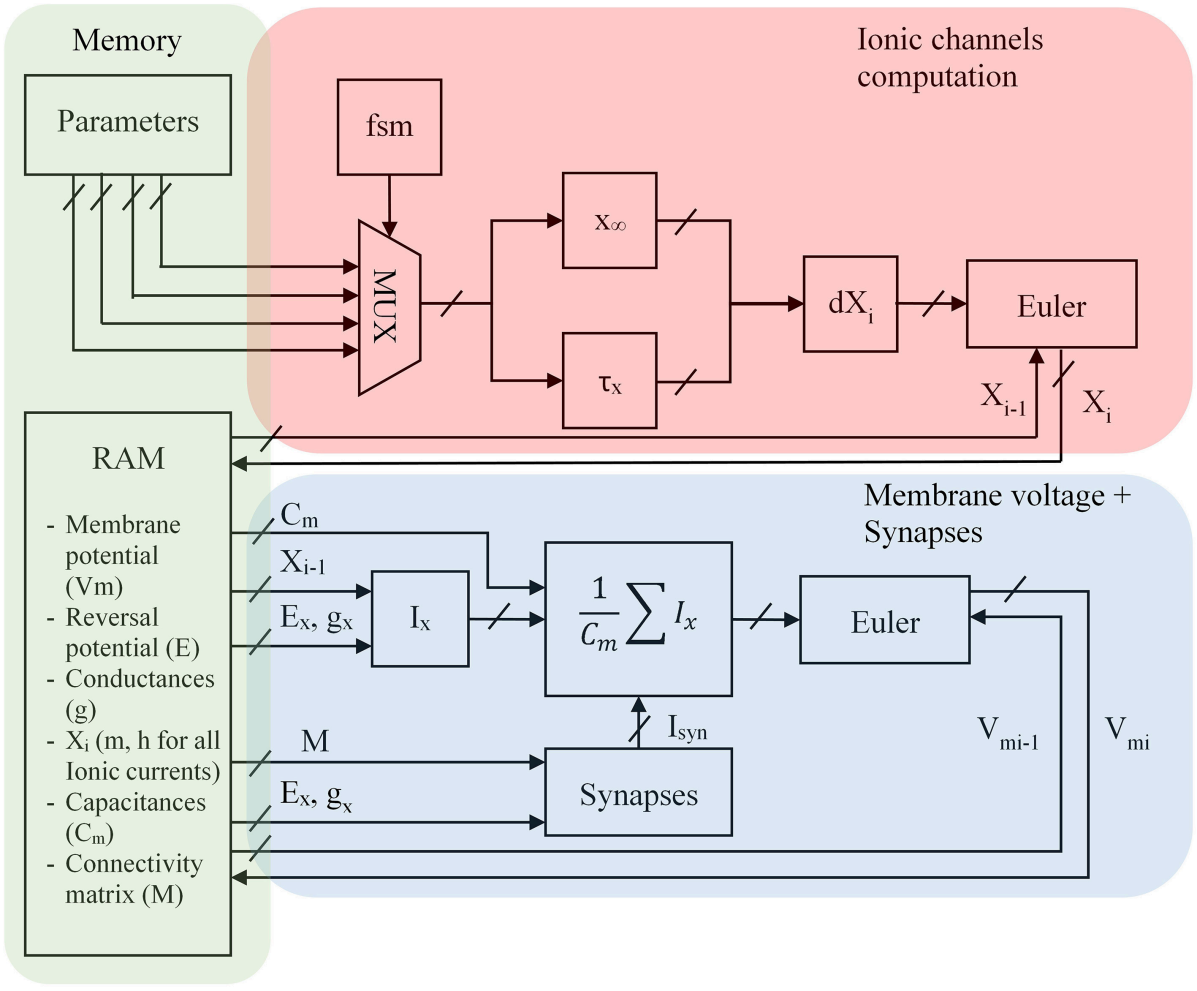
FPGA architecture for the optimized Hodgkin-Huxley equations where X_i_ represents m and h for the different ion currents, E_x_ the equilibrium voltage, g_x_ the conductance value, i is the discretized time, x_∞_ and τ_x_ the equations presented in the previous part, I_x_ the computation of the ionic current where x represents K, Na, P, CaL or CaT, Cm the membrane capacitance; Parameters are shown in part Alternative Equations; FSM is a Finite State Machine and selects every cycle a ionic channel to compute. The red part is the computation of ionic channel. The blue part is the computation of the neuron. The green part is the store of parameters in memory blocks.

The architecture has been designed for taking few resources and to compute a maximum of neurons. Computation of exponentials, hyperbolic tangents and divisions are low resource modules in the FPGA. This novel architecture uses the same computation module to calculate all the ionic channels using one hyperbolic tangent and one hyperbolic cosine for all the neurons computation and is presented in Figure 7.

Every cycle a Finite State Machine (FSM) selects different parameters (see part Alternative Equations) for the computation of x_∞_ and τ_x_ according to the ionic channel which is then stored into a Random Access Memory present in the FPGA. A parallel equation is solved taking the I – 1 value of ionic channel to compute the ionic current and then the membrane voltage. The synapses are selected by a connectivity matrix which contains conductances. The V_post_ value can be computed by the product between the transposed connectivity matrix and the voltage membrane (V_pre_) vector.

**Figure 8:**
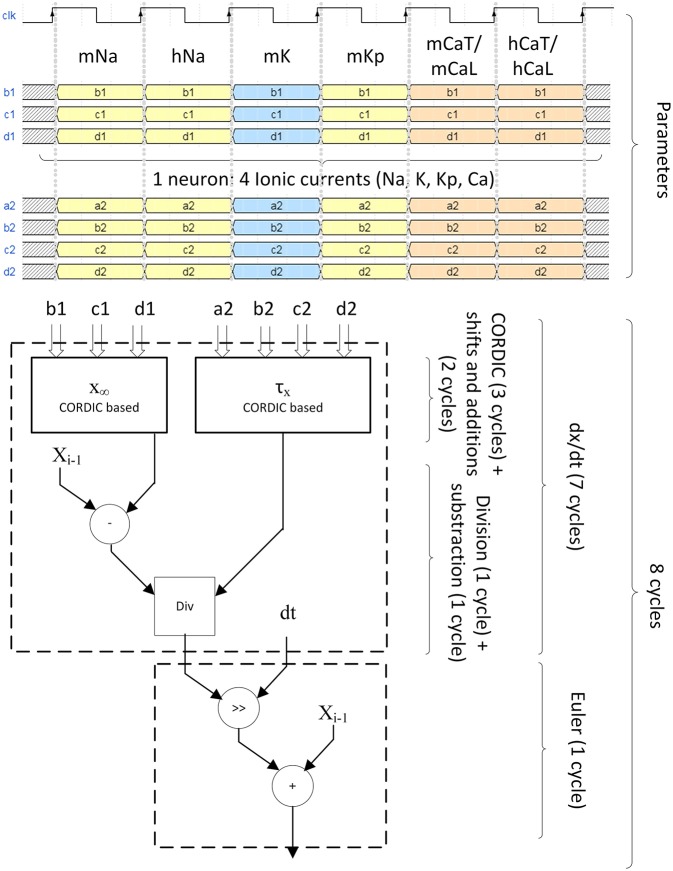
Pipeline structure of the ionic gate computation. Four different ionic currents composes the computation of one neuron (INa, IK, IKp, ICaL for IB, ICaT for LTS). For INa, ICaL, and ICaT, they are divided in two states called m and h which are the probability of opening and closing of the channel. Six clock cycles are needed to send of all probability parameters. Eight clock cycles are needed to compute the ionic gate equation. The computation of x∞ and τx uses CORDIC algorithm for hyperbolic functions. The differential equation is solved using Euler technique.

All the computation is pipeline-based. Figure 8 explains the timing of computation for the 4 ionic channels to compute one neuron.

## Result

### FPGA Resources

The optimized Hodgkin-Huxley equations are calculated using a dt of 2^−5^ ms during eight cycles (Figure 8) at 100 MHz. It corresponds to a computation time of **80 ns** for the calculation of one neuron using the proposed pipelined architecture. Then the membrane voltage of a new neuron can be computed every cycle because of the pipelined architecture. The maximum number of neurons is determined by the computation of the ionic channel due to its long calculation period (eight cycles or 80 ns), and it must follow the rule described by Equation (29).

(29) $$\begin{array}{l} 8.T_{clk}+N.6.T_{clk}\leq dt \end{array}$$

where **T**_**clk**_ is the period of the clock frequency of the FPGA (100 MHz), **dt** the calculation step (2^−5^ ms), **N** the number of neurons. The constant **8** corresponds to the number of cycles needed to compute one ionic channel and the value **6** represents the clock cycles which are needed for the sending of all four ionic currents. Then, the maximum number of neurons of one core is 500 and could be increase to 15,000 neurons if we use all the resources (30 cores) which is limited by the number of DSP. In this implementation, we reduced the number of DSP for one core compared to other architecture proposed in the state of the art.

The FPGA implementation resources are presented according to the number of neurons in which shows good performances of the optimized equations and architecture compared to other architectures.

Table 5 demonstrates the efficiency of our implementation. The work of Bonabi et al. (2014) and Akbarzadeh-Sherbaf et al. (2018) proposed implementation of three ionic currents (Na, K and Leak) which reproduce Fast Spiking (FS) neuron family. The number of neurons for one core computation was multiplied by 6.9 compared to Bonabi et al. (2014) and by 2 compared to Akbarzadeh-Sherbaf et al. (2018). Often, the critical point in FPGA implementation is the number of DSP available in FPGA platform. Our work reduced by 69.5 compared to Bonabi et al. (2014) and by 4.35 compared to Akbarzadeh-Sherbaf et al. (2018).

**Table 5:** Resource table according to the number of neurons N, the frequency F and the resources in LUT, Flip-Flops (FF) and DSPs; In blue, our work and in red, implementation of HH on FPGA presented in other publications; The maximum number of synapses is considered to be N^2^ which represent an all to all connection case; The results of this works includes into the resources the DAC handle; (Akbarzadeh-Sherbaf et al., 2018) show a maximum of 5,120 neurons in real-time with 10 cores.

	**Original equations**	**One core (only FS)**	**One core (FS, RS, IB, LTS) + N^2^ synapses + synaptic noise**	**30 cores (FS, RS, IB, LTS) + N^2^ synapses + synaptic noise**	**(Bonabi et al., 2014)**	**4 cores (only FS) (Akbarzadeh-Sherbaf et al., 2018)**
N	150	1,034	500	15,000	150	2,048
F (MHz)	100	100	100	100	63.386	58.8
LUT	169,003	4,735	5,551	167,346	86,032	46,045
FF	65,059	1,552	2,360	71,608	30,528	4,606
DSP	332	16	28	840	1,112	280

*It means 512 neurons for one core. Our implementation can multiply by 2 this number with reducing the resources especially DSP numbers*.

Our implementation including FS, RS, LTS IB neurons, synapses and synaptic noise can simulate 500 neurons in real-time for one core of computation.

### Neuron Validation

**Figure 9:**
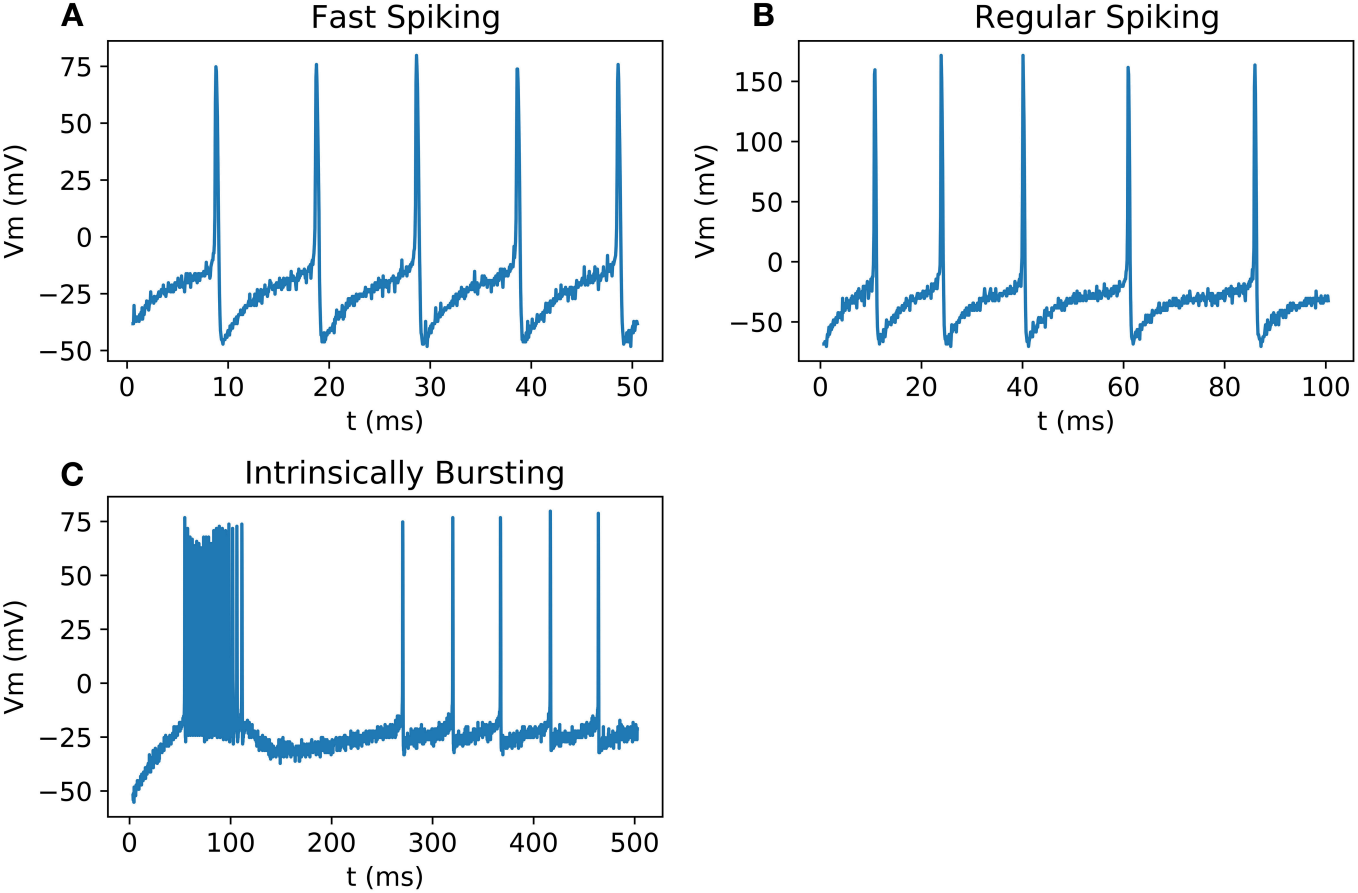
Signals measured from DAC on scope; **(A)** FS with a stimulation current of 0.5 nA; **(B)** RS with a stimulation current of 0.75 nA; **(C)** IB with a stimulation current of 0.15 nA.

All the neurons can be output on an oscilloscope and show similar behavior as the biological neurons presented in Pospischil et al. (2008) and as Matlab simulations (Figure 9). Because of the low precision of the DAC compared to the amplitude of an AP, the result has been multiplied by a factor and an offset has been added.

Figure 9 shows the validation of our neuron families with real-time output in oscilloscope. Spike timing and shape are the same compared to Matlab simulation and Vivado simulations.

### Stochastic Neuron Validation

**Figure 10:**
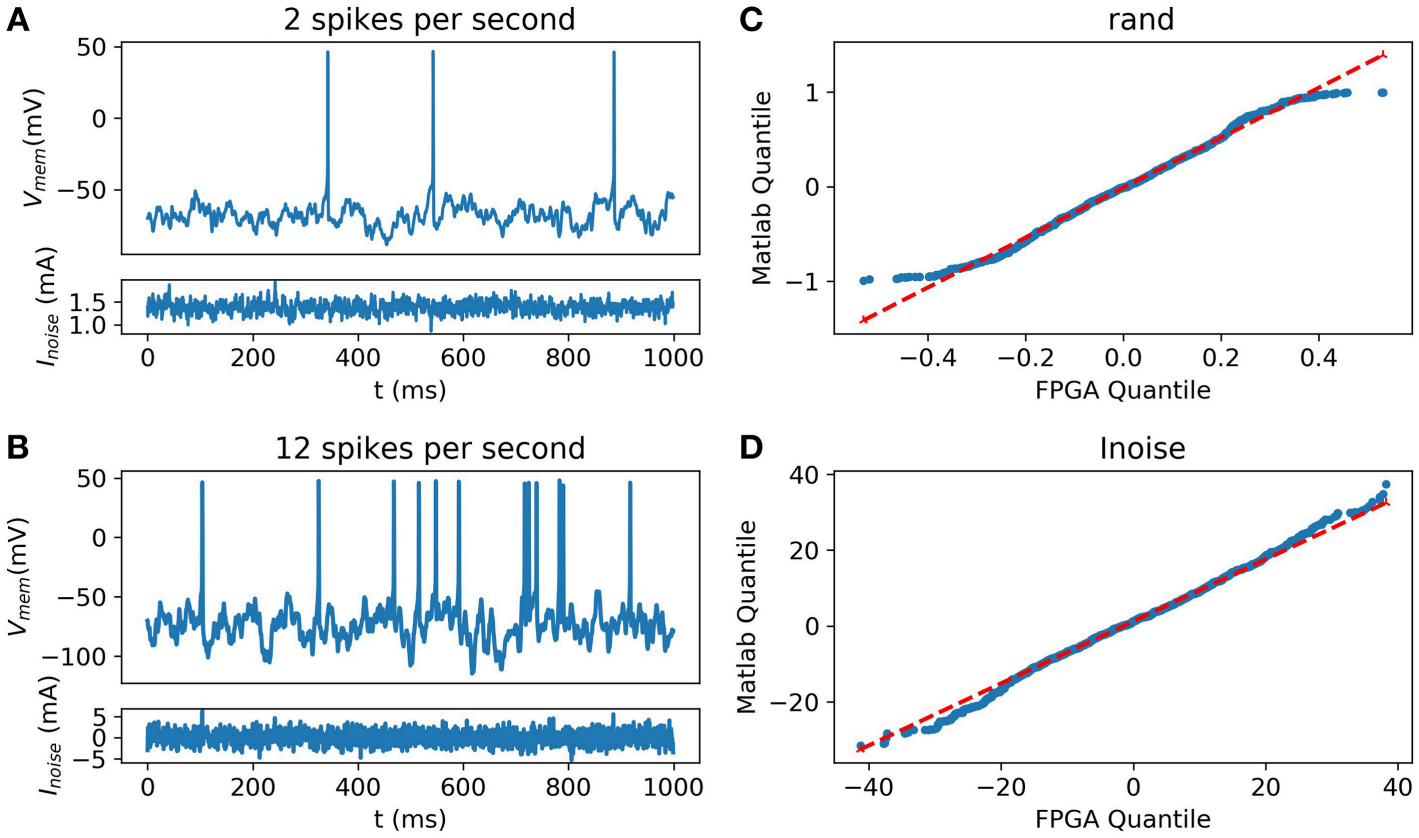
Synaptic noise validation. All blue data are from the scope **(A)** Random and Spontaneous activities raised by the noise using θ = 1, μ = 0.1, and σ = 1.05 **(B)** Random and Spontaneous activities raised by the noise using θ = 1, μ = 0.1, and σ = 2.2; **(C)** Quantile-Quantile (QQ) plot showing the similarities between the random function into Matlab and from the FPGA; **(D)** (QQ) plot showing similar behavior between the current representing the noise computed on Matlab and generated by the FPGA.

Using the HH implementation on the FPGA coupled with the digital implementation of the OU process (Grassia et al., 2016), the system shows some random and spontaneous activities (Figure 10).

Inter-spike Interval (ISI) has been determined around 10.4 ± 4.5 spikes per second in Neocortical cells of rats by Nawrot et al. (2007) and 2.5 spikes per second in cerebral cortex neurons of cats (Webb and Burns, 1976). Spike rates of spontaneous activity are variable in living cells. Thus, the equation can be tuned to fit with the biological behavior. The probability distribution between the noise from Matlab and the one implemented into the FPGA was compared using the Quantile-Quantile (QQ) plot showing strong correlation and similarities between experimental data and data obtained using hardware implementation.

Figure 10 describes the validation of synaptic noise with oscilloscope outputs. The stimulation current which includes synaptic noise allows stochastic behavior of the neuron that mimics closer to biological behavior. This dynamic stimulation current also validates the dynamic behavior of the neuron. Spontaneous biological activity can be reproduced thanks to this synaptic noise.

### Neural Network Validation

**Figure 11:**
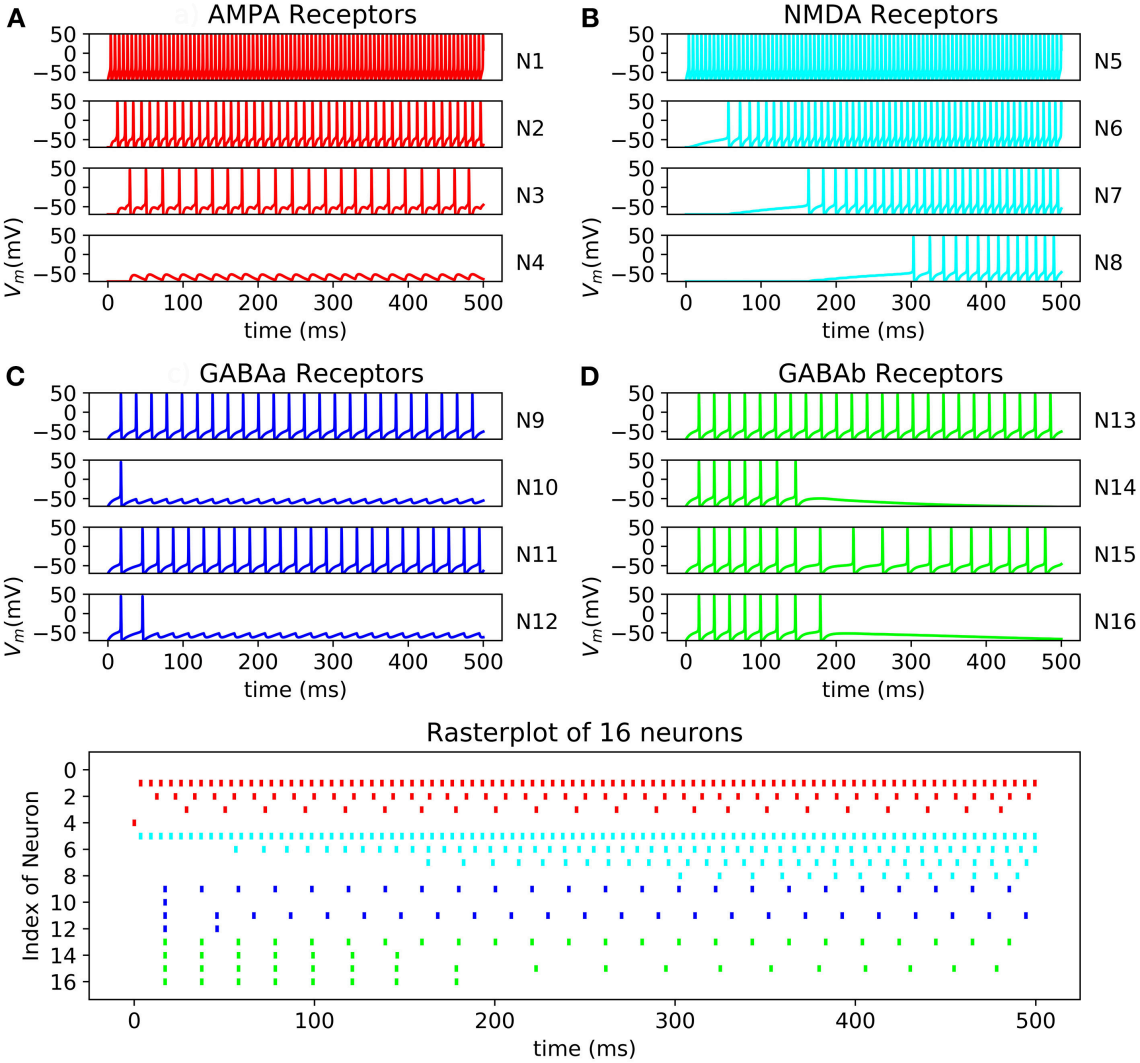
Raster plot of 16 neurons with their respective oscilloscope output **(A)** N13->N14->N15->N16 with GABAb synapse showing a longer response of the inhibition than GABAa; **(B)** N9->N10 and N11->N12 showing fast inhibition of N10 and N12; **(C)** N5->N6->N7->N8 by NMDA synapses demonstrated that N5 stimulate N6 which stimulate N6; **(D)** N1->N2->N3->N4 by AMPA synapses showing faster response of excitation than NMDA synapses, synaptic conductance of the synapse connecting N3 to N4 is too small resulting in a lack of excitation of N4 and no spike.

All the neurons can be connected to each other using a connectivity matrix representing how the neurons are connected. The behavior of AMPA, NMDA, GABAa, and GABAb can be reproduced (Figure 11). The raster plot represents the effect of excitatory synapses and inhibitory synapses on neurons through 16 cells. The system is able to compute 500 neurons in one core or 15,000 using 30 core which can be output through a raster plot.

This platform shows the possibility of computed cortical network using different classes of neurons and different receptors. All the bio-physical parameters can be modified using a software developed in Python 3.6 to communicate through Universal Asynchronous Receiver Transmitter (UART) with the FPGA. The developed solution for communication is able to send the needed parameters and to receive raster plot. Thus, such a system could be used to simulate complex cortical network or to perform bio-hybrid experiments.

## Conclusion and perspectives

A biomimetic neural network has been designed including synapses and synaptic noise. This real-time system is tunable and mimics the activities of four main important cortical neuron types and four synaptic receptor types. The optimization of the Hodgkin-Huxley equations using hyperbolic function and power of two parameters allows the use of large scale with low resources of biomimetic neurons. Also, algorithms, such as non-restoring division, Euler method, and CORDIC make the system faster in terms of frequency in low power consumption. Thus, our results show a strong correlation between the hardware implementation of our optimized solution and the original model. On the other hand, bifurcation analysis describes stability and similarity to dynamic of our model compared to the original one. The performances are compared to other works and demonstrate that this system goes further than the state of the art in terms of features and bio-plausibility. Our architecture allows us to compute and output real-time neural network activities for bio-hybridization experiments. Moreover, all the HH, synapses and noise parameters can be modified through a UART connection using a software which has been developed for this purpose. Output tunable real-time neuron activities is not possible using conventional computing with such a number of neurons and ionic currents. UART is a good and simple solution for as small number of neurons. However, the number of parameters increases with the number of neurons letting the possibility of using TCP/IP protocol for bigger networks. This article also describes different tools for studying the variation that could occurs into the HH model during the optimization. The bifurcation analysis validates the similarity in the dynamic of the new system compared to the original one. Also, the cross-correlation gives good correlation coefficient and shows a small phase between the optimized AP and the original one. Measuring the correlation between two signals gives a “r” value that can be analyzed as strong correlated when close to 1, but how close do the system need to be able to communicate with living cells?

The aim of this work was primarily to provide a scalable and tunable platform allowing the study of neurological diseases. By connecting a real-time neural network to living cells, it will be possible to observe the effect of the different biophysical parameters on a biological network. Using an artificial neural network affected by a disease, the effect of the affliction on the living cell can be observed. This work is the first step toward new ways of studying the brain and understanding the possibilities of connecting nerve cells with the machine.

The next step is to reproduce a larger neural network like pyramidal layer structure and to connect this biomimetic SNN with neuron culture or brain organoids (Kawada et al., 2017). These closed-loop bidirectional experiments can test some therapeutic stimulation protocols. Another point is to increase the degree of precision by designing multi-compartmental neurons and give a real-time tool to neuroscientist to simulate their neuron structure with different parameters. To conclude, this biomimetic SNN platform can provide better solutions for biomedical application especially in neuroprosthesis (Buccelli et al., 2019) and therapeutics protocol for neurological diseases.

Another way to explore is linked to Artificial Intelligence (AI). We will implement in the next future some learning rules like STDP to perform AI algorithm (Zhang et al., 2018) like pattern recognition. This biomimetic SNN can reproduce the spike timing but also the shape of neurons. Debanne et al. (2013) indicates that subthreshold variation in the presynaptic membrane potential also determines spike-evoked transmission and that membrane voltage might modulate neurotransmitter release. Brette (2015) discusses about the difference between rate-based or spiked-based computation and the possible real neural coding used in the brain. We think that being closer to the biology will lead to better results in AI algorithms using less number of neurons and hidden layers. In more long term future, it will allow neurobiohybrid experiments using biological intelligence and AI in the same time for maximizing the efficiency.

## Author Contributions

FK modeled and implemented the spiking neural network. FG studied the noise and the bifurcation analysis. All the authors edited the manuscript. SS and TL acquired the funding and supervised the whole study.

### Conflict of Interest Statement

The authors declare that the research was conducted in the absence of any commercial or financial relationships that could be construed as a potential conflict of interest.

## Footnotes

^1^http://www.fz-juelich.de/ias/jsc/EN/Expertise/Supercomputers/JUQUEEN/Configuration/Configuration_node.html

^2^http://www.riken.jp/en/pr/press/2013/20130802_1/

## References

[B1] Akbarzadeh-Sherbaf K. Abdoli B. Safari S. Vahabie A.-H. (2018). A scalable FPGA architecture for randomly connected networks of hodgkin-huxley neurons. Front. Neurosci. 12:698 10.3389/fnins.2018.00698 30356803 PMC6190648

[B2] Alle H. Geiger J. R. P. (2006). Combined analog and action potential coding in hippocampal mossy fibers. Science 311, 1290–1293. 10.1126/science.1119055 16513983

[B3] Ambroise M. Levi T. Joucla S. Yvert B. Saïghi S. (2013). Real-time biomimetic central pattern generators in an FPGA for hybrid experiments. Front. Neurosci. 7:215 10.3389/fnins.2013.00215 24319408 PMC3836270

[B4] Andraka R. (1998). A survey of CORDIC algorithms for FPGA based computers, in ACM/SIGDA International Symposium on Field Programmable Gate Arrays – FPG. Monterey, US.

[B5] Arthur J. Merolla P. Akopyan F. Alvarez R. Cassidy A. Chandra S. (2012). Building block of a programmable neuromorphic substrate: a digital neurosynaptic core,. in Proceedings of the International Joint Conference on Neural Networks, Brisbane, QLD.

[B6] Bareket-Keren L. Hanein Y. (2012). Carbon nanotube-based multi electrode arrays for neuronal interfacing: progress and prospects. Front. Neural Circuits 6:122. 10.3389/fncir.2012.00122 23316141 PMC3540767

[B7] Binczak S. Jacquir S. Bilbault J. M. Kazantsev V. Nekorkin V. (2006). Experimental study of electrical fitzhugh-nagumo neurons with modified excitability. Neural Net. 19, 684–693. 10.1016/j.neunet.2005.07.011 16182512

[B8] Bonabi S. Y. Asgharian H. Safari S. Ahmadabadi M. N. (2014). FPGA implementation of a biological neural network based on the Hodgkin-Huxley neuron model. Front. Neurosci. 8:379 10.3389/fnins.2014.00379 25484854 PMC4240168

[B9] Bonifazi P. Difato F. Massobrio P. Breschi G. L. Pasquale V. Levi T.. (2013). *In vitro* large-scale experimental and theoretical studies for the realization of bi-directional brain-prostheses. Front. Neural Circuits 7:40 10.3389/fncir.2013.00040 23503997 PMC3596784

[B10] Brette R. (2015). Philosophy of the spike: rate-based vs. spike-based theories of the brain, Front. Syst. Neurosci. 9:151 10.3389/fnsys.2015.00151 26617496 PMC4639701

[B11] Brette R. Gerstner W. (2005). Adaptive exponential integrate-and-fire model as an effective description of neuronal activity. J. Neurophysiol. 94, 3637–3642. 10.1152/jn.00686.2005 16014787

[B12] Broccard F. D. Joshi S. Wang J. Cauwenberghs G. (2017). Neuromorphic neural interfaces: from neurophysiological inspiration to biohybrid coupling with nervous systems. J. Neural Eng. 14:041002. 10.1088/1741-2552/aa67a9 28573983

[B13] Buccelli S. Bornat Y. Colombi I. Ambroise M. Martines L. Pasquale V. (2019). A neuroprosthetic system to restore neuronal communication in modular networks. bioRxiv 514836. 10.1101/514836

[B14] Capogrosso M. Milekovic T. Borto D. Wagner F. Moraud E. M. Mignardot J. B.. (2016). A brain–spine interface alleviating gait deficits after spinal cord injury in primates. Nature 539, 284–288 10.1038/nature20118 27830790 PMC5108412

[B15] Cassidy A. Andreou A. Georgiou J. (2011). Design of a one million neuron single FPGA neuromorphic system for real-time multimodal scene analysis, in 45th Annual Conference on Information Sciences and Systems, CISS 2011. Baltimore, US.

[B16] Chou Z. Lim J. Brown S. Keller M. Bugbee J. Broccard F. D.. (2014). Bidirectional neural interface: closed-loop feedback control for hybrid neural systems. Conf. Proc. IEEE. Eng. Med. Biol. Soc. 2015, 3949–3952. 10.1109/EMBC.2015.7319258. 26737158

[B17] Cohen M. Kohn A. (2013). Measuring and interpreting neuronal correlations. Nat. Neurosci. 14, 811–819. 10.1038/nn.2842 21709677 PMC3586814

[B18] Debanne D. Bialowas A. Rama S. (2013). What are the mechanisms for analogue and digital signalling in the brain?. Nat. Rev. Neurosci. 14, 63–69 10.1038/nrn3361 23187813

[B19] Destexhe A. Mainen Z. F. Sejnowski T. J. (1998). Kinetic Models of Synaptic Transmission. Methods I Neuronal Modeling. Cambridge, MA: MIT Press, 1–25.

[B20] Destexhe A. Rudolph M. Fellous J.-M. Sejnowski T. J. (2001). Fluctuating synaptic conductances recreate *in vivo*-like activity in neocortical neurons. Neuroscience 107, 13–24. 10.1016/S0306-4522(01)00344-X 11744242 PMC3320220

[B21] Fernandez-Vargas J. Pfaff H. Rodríguez F. Varona P. (2013). Assisted closed-loop optimization of SSVEP-BCI efficiency. Front. Neural Circuits 7:27 10.3389/fncir.2013.00027 23443214 PMC3580891

[B22] Furber S. Lester D. Plana L. Garside J. Painkras E. Temple S. (2013). Overview of the SpiNNaker system architecture. IEEE Trans. Comput. 62,2454–2467. 10.1109/TC.2012.142

[B23] George S. Hasler J. Koziol S. Nease S. Ramakrishnan S. (2013). Low power dendritic computation for wordspotting. Low Power Electron. Appl. 3:73–98 10.3390/jlpea3020073

[B24] Gewaltig M.-O. Diesmann M. (2007). NEST (NEural Simulation Tool). Scholarpedia 2:1430 10.4249/scholarpedia.1430

[B25] Goodman D. F. M. Brette R. (2009). The Brian simulator. Front. Neurosci. 3, 192–197. 10.3389/neuro.01.026.2009 20011141 PMC2751620

[B26] Grassia F. Buhry L. Levi T. Tomas J. Destexhe A. Saïghi S. (2011). Tunable neuromimetic integrated system for emulating cortical neuron models. Front. Neurosci. 5:134 10.3389/fnins.2011.00134 22163213 PMC3233664

[B27] Grassia F. Kohno T. Levi T. (2016). Digital hardware implementation of a stochastic two-dimensional neuron model. J. Physiol. Paris 110, 409–416 10.1016/j.jphysparis.2017.02.002 28237321

[B28] Grassia F. Lévi T. Saïghi S. Kohno T. (2012). Bifurcation analysis in a silicon neuron. Artificial Life Robot. 17:53–58 10.1007/s10015-012-0016-6

[B29] Gutnick M. Mody I. (2012). The Cortical Neuron. Oxford Scholarship 33–51

[B30] Hansel D. Mato G. Meunier C. (1993). Phase dynamics for weakly coupled hodgkin–huxley neurons. Europhys. Lett. 23:367–372 10.1209/0295-5075/23/5/011

[B31] Hasler P. Koziol S. Farquhar E. Basu A. (2007). Transistor channel dendrites implementing hmm classifiers, in IEEE International Symposium on Circuits and Systems. New Orleans, US.

[B32] Hassard B. (1978). Bifurcation of periodic solutions of the Hodgkin-Huxley model of the squid giant axon. J. Theor. Biol. 71, 401–420 10.1016/0022-5193(78)90168-6 642538

[B33] Hassard B. Kazarinoff N. D. Wan Y. H. (1981). Theory and Appli-Cations of Hopf Bifurcation. Cambridge: Cambridge University Press.

[B34] Higham D. J. (2001). An algorithmic introduction to numerical simulation of stochastic differential equations. SIAM Rev. 43, 525–546. 10.1137/S0036144500378302

[B35] Hines M. L. Carnevale N. T. (2001). N.T. NEURON: a tool for neuroscientists. Neuroscientist 7, 123–135 10.1177/107385840100700207 11496923

[B36] Hochberg L. R. Bacher D. Jarosiewicz B. Masse N. Y. Simeral J. D. Vogel J.. (2012). Reach and grasp by people with tetraplegia using a neurally controlled robotic arm. Nat. Methods 485, 372–375. 10.1038/nature11076 22596161 PMC3640850

[B37] Hodgkin A. L. (1948). The local electric changes associated with repetitive action in a non medullated axon. J. Physiol. 107, 165–181 10.1113/jphysiol.1948.sp004260 16991796 PMC1392160

[B38] Hodgkin A. L. Huxley A. F. (1952). A quantitative description of membrane current and its applications to conduction and excitation in nerve. J. Physiol. 117, 500–544. 10.1113/jphysiol.1952.sp004764 12991237 PMC1392413

[B39] Ide J. S. Cappabianco F. A. Faria F. A. Li C. R. (2017). Detrented Partial Cross Correlation for Brain Connectivity Analysis, in 31st Conference on Neural Information Processing Systems. NIPS 2017, Long Beach, US

[B40] Indiveri G. Fusi S. (2007). Spike-based learning in VLSI networks of integrate-and fire neurons, IEEE International Symposium on Circuits and Systems, New Orleans, US, 3371–3374.

[B41] Indiveri G. Inares-Barranco B. Hamilton T. J. Van Schaik A. Etienne-Cummings R. Delbruck T.. (2001). Neuromorphic silicon neuron circuits. Front. Neurosci. 5:73. 10.3389/fnins.2011.00073 21747754 PMC3130465

[B42] Izhikevich E. M. (2000). Neural excitability, spiking and bursting. IJBC 10, 1171–1266. 10.1142/S0218127400000840

[B43] Izhikevich E. M. (2003). Simple model of spiking neurons. IEEE Trans. Neural Net. 14, 1569–1572. 10.1109/TNN.2003.820440 18244602

[B44] Jordan J. Ippen T. Helias M. Kitayama I. Mitsuhisa S. Igarashi J. (2018). Extremely scalable spiking neural network simulation code: from laptops to exascale computers. Front. Neuroinformatics 12:2 10.3389/fninf.2018.00002 PMC5820465 29503613

[B45] Joucla S. Ambroise M. Levi T. Lafon T. Chauvet P. Saïghi S.. (2016). Generation of locomotor-like activity in the isolated rat spinal cord using intraspinal electrical microstimulation driven by a digital neuromorphic CPG. Front. Neurosci. 10:67 10.3389/fnins.2016.00067 27013936 PMC4779903

[B46] Jung R. Brauer E. Abbas J. (2001). Real-time interaction between a Neuromorphic Electronic Circuit and the Spinal Cord. IEEE Trans. Neural Syst. Rehabil. Eng. 9, 319–326 10.1109/7333.948461 11561669 PMC4677037

[B47] Kawada J. Kaneda S. Kirihara T. Maroof A. Levi T. Eggan K.. (2017). Generation of a motor nerve organoid with human stem cell-derived neurons. Stem Cell Rep. 9, 1441–1449. 10.1016/j.stemcr.2017.09.021 29107592 PMC5831012

[B48] Kohno T. Sekikawa M. Li J. Nanami T. Aihara K. (2016). Qualitative-modeling-based silicon neurons and their networks. Front. Neurosci. 10, 1–16. 10.3389/fnins.2016.00273 27378842 PMC4908299

[B49] Kunkel S. Schmidt M. Eppler J. M. Plesser H. E. Masumoto G. Igarashi J.. (2014). Spiking network simulation code for petascale computers. Front. Neuroinformatics 8:78. 10.3389/fninf.2014.00078 25346682 PMC4193238

[B50] Le Masson G. Renaud-Le Masson S. Debay D. Bal T. (2002). Feedback inhibition controls spike transfer in hybrid thalamic circuits. Nature 417, 854–858 10.1038/nature00825 12075353

[B51] Levi T. Bonifazi P. Massobrio P. Chiappalone M. (2018c). Closed-loop systems for next-generation neuroprostheses. Front. Neurosci. 12, 7–9. 10.3389/fnins.2018.00026 29483859 PMC5816068

[B52] Levi T. Fujii T. (2016). Microfluidic neurons, a new way in neuromorphic engineering? Micromachines 7:146 10.3390/mi7080146 30404317 PMC6189925

[B53] Levi T. Khoyratee F. Saighi S. Ikeuchi Y. (2018a). Digital implementation of Hodgkin–Huxley neuron model for neurological diseases studies. J. Artif. Life Robot. Springer Nat. 23, 10–14 10.1007/s10015-017-0397-7

[B54] Levi T. Lewis N. Tomas J. Saighi S. Renaud S. Bornat Y. (2008). Neuromimetic integrated circuits, VLSI circuits for biomedical applications, artech house. Iniewski K 241–264.

[B55] Levi T. Nanami T. Tange A. Aihara K. Kohno T. (2018b). Development and applications of biomimetic neuronal networks toward brainmorphic artificial intelligence. IEEE Trans. Circuits Syst. 65, 577–581 10.1109/TCSII.2018.2824827

[B56] Levitan H. Segundo J. P. Moore G. P. Perkel D. H. (1968). Statistical analysis of membrane potential fluctuations. Biophys. J. 8, 1256–1274. 10.1016/S0006-3495(68)86554-3 4301347 PMC1367693

[B57] Liu S. Douglas R. (2004). Temporal coding in a silicon network of integrate-and fire neurons. IEEE Trans. Neural Net. 15, 1305–1314. 10.1109/TNN.2004.832725 15484903

[B58] Mahowald M. Douglas R. (1991). A Silicon neuron. Nature 1991, 515–518 10.1038/354515a0 1661852

[B59] Manwani A. Koch C. (1999). Detecting and estimating signals in noisy cable structures. I: neuronal noise sources. Neural Comput. 11, 1797–1829. 10.1162/089976699300015972 10578033

[B60] Markram H. (2012). The human brain project. Sci. Am. 306, 50–55 10.1038/scientificamerican0612-50 22649994

[B61] Merolla P. A. Arthur J. V. Alvarez-Icaza R. Cassidy A. S. Sawada J. Akopyan F.. (2014). A million spiking-neuron integrated circuit with a scalable communication network and interface. Science 345:668 10.1126/science.1254642 25104385

[B62] Nanami T. Kohno T. (2016). Simple cortical and thalamic neuron models for digital arithmetic circuit implementation. Front. Neurosci. 10, 1–12 10.3389/fnins.2016.00181 27242397 PMC4865656

[B63] Natarajan A. Hasler J. (2018). Hodgkin-huxley neuron and fpaa dynamics. IEEE Trans Biomed Circuits Syst. 12, 918–926 10.1109/TBCAS.2018.2837055 30010587

[B64] Nawrot M. P. Boucsein C. Rodriguez-Molina V. Aertsen A. Grün S. Rotter S. (2007). Serial interval statistics of spontaneous activity in cortical neurons *in vivo* and *in vitro*. Neurocomputing 70, 1717–1722. 10.1016/j.neucom.2006.10.101

[B65] Nazari S. Faez K. Amiri M. Karami E. (2015). A digital implementation of neuron-astrocyte interaction for neuromorphic applications. Neural Net. 66, 79–90 10.1016/j.neunet.2015.01.005 25814323

[B66] Nicolelis M. A. L. Lebedev M. A. (2009). Principles of neural ensemble physiology underlying the operation of brain-machine interfaces. Nat. Rev. Neurosci. 10, 530–540 10.1038/nrn2653 19543222

[B67] Nishimura Y. Perlmutter S. Fetz E. (2013). Restoration of upper limb movement via artificial corticospinal and musculospinal connections in a monkey with spinal cord injury. Front. Neural Circuits 7:57 10.3389/fncir.2013.00057 23596396 PMC3622884

[B68] Obermann S. F. Flynn M. J. (1997). Division algorithms and implementations. IEEE Trans. Comput. 46, 833–854 10.1109/12.609274

[B69] Opris I. Fuqua J. Huettl P. Gerhardt G. Berger T. Hampson R.. (2012). Closing the loop in primate prefrontal cortex: inter-laminar processing. Front. Neural Circuits 6:88 10.3389/fncir.2012.00088 23189041 PMC3504312

[B70] Osorio R. R. (2016). Pipelined FPGA implementation of numerical integration of the Hodgkin-Huxley model, in 2016 IEEE 27th International Conference on Application-specific Systems, Architectures and Processors (ASAP). London, United Kingdom, 202–206

[B71] Pimashkin A. Gladkov A. Mukhina I. Kazantsev V. (2013). Adaptive enhancement of learning protocol in hippocampal cultured networks grown on multielectrode arrays. Front. Neural Circuits 7:87 10.3389/fncir.2013.00087 23745105 PMC3662887

[B72] Pospischil M. Toledo-Rodriguez M. Monier C. Powkowska Z. Bal T. Frégnacn Y.. (2008). Minimal Hodgkin-Huxley type models for different classes of cortical and thalamic neurons. Biol. Cybern. 99:427–441 10.1007/s00422-008-0263-8 19011929

[B73] Potter S. El Hady A. Fetz E. (2014). Closed-loop neuroscience and neuroengineering. Front. Neural Circuits 8, 2013–2015. 10.3389/fncir.2014.00115 25294988 PMC4171982

[B74] Qiao N. Mostafa H. Corradi F. Osswald M. Stefanini F. Sumislawska D.. (2015). A reconfigurable on-line learning spiking neuromorphic processor comprising 256 neurons and 128K synapses. Front. Neurosci. 9:141 10.3389/fnins.2015.00141 25972778 PMC4413675

[B75] Rast A. D. Partzsch J. Mayr C. Schemmel L. Hartmann S. Plana L. A. (2013). A location-independent direct link neuromorphic interface, in Proceedings of the International Joint Conference on Neural Networks, Dallas, US, 1967–1974.

[B76] Renaud S. Tomas J. Bornat Y. Daouzli A. Saïghi S. (2007). Neuromimetic ICs with analog cores: an alternative for simulating spiking neural networks, IEEE International Symposium on Circuits and Systems, New-Orleans, USA 3355–3358.

[B77] Rice K. Bhuiyan M. Taha T. Vutsinas C. Smith M. (2009). FPGA Implementation of Izhikevich Spiking Neural Networks for Character Recognition, in International Conference on Reconfigurable Computing and FPGAs, Cancun, Mexico 451–456.

[B78] Rinzel J. Ermentrout G. B. (1989). Analysis of Neural Excitability and Oscillations. Methods in Neural Engineering. Cambridge: MIT Press.

[B79] Robinson J. Jorgolli M. Park H. (2013). Nanowire electrodes for high-density stimulation and measurement of neural circuits. Front. Neural Circuits 7:38. 10.3389/fncir.2013.00038 23486552 PMC3594763

[B80] Sabarad J. Kestur S. Park M. Dantara D. Narayanan V. Chen Y. (2012). A reconfigurable accelerator for neuromorphic object recognition, in Proceedings of the Asia and South Pacific Design Automation Conference. ASP-DAC, Sidney, Australia 813–818.

[B81] Schemmel J. Bruderle D. Meier K. Ostendorf B. (2007). Modeling synaptic plasticity within networks of highly accelerated I&F neurons, in IEEE International Symposium on Circuits and Systems (New Orlean), 3367–3370.

[B82] Serb A. Corna A. George R. Khiat A. Rocchi F. Reato M. (2017). A geographically distributed bio-hybrid neural network with memristive plasticity. arXiv: 1709.04179

[B83] Seth A. K. Barrett A. B. Barnett L. (2015). Granger causality analysis in neurosicence and neuroimaging. J. Neurosci. 35, 3293–3297. 10.1523/JNEUROSCI.4399-14.2015 25716830 PMC4339347

[B84] Shahdoost S. Frost S. Acker G. van Dejong S. Barbay S. Nudo R.. (2014). Towards a miniaturized brain-machine-spinal cord interface (bmsi) for restoration of function after spinal cord injury, in 36th Annual International Conference of the IEEE Engineering in Medicine and Biology Society. Chicago, IL. 10.1109/EMBC.2014.6943634 25570002

[B85] Sorensen M. DeWeerth S. Cymbalyuk G. Calabrese R. (2004). Using a hybrid neural system to reveal regulation of neuronal network activity by an intrinsic current. J. Neurosci. 24, 5427–5438. 10.1523/JNEUROSCI.4449-03.2004 15190116 PMC6729308

[B86] Stein R. B. Gossen E. R. Jones K. E. (2005). Neuronal variability: noise or part of the signal? Nat. Rev. Neurosci. 6, 389–397. 10.1038/nrn1668 15861181

[B87] Sutter G. Deschamps J. (2009). High speed fixed point dividers for FPGAs, in International Conference on Field Programmable Logic and Applications (Pragues).

[B88] Tuckwell H. C. (1988). Introduction to Theoretical Neurobiology. Cambridge, UK: Cambridge University Press.

[B89] Tuckwell H. C. Wan F. Y. M. Rospars J.-P. (2002). A spatial stochastic neuronal model with Ornstein–Uhlenbeck input current. Biol. Cybern. 86, 137–145. 10.1007/s004220100283 11911115

[B90] Van Albada S. J. Rowley A. G. Senk J. Hopkins M. Schmidt M. Stokes A. B.. (2018). Performance comparison of the digital neuromorphic hardware SpiNNaker and the neural network simulation software NEST for a full-scale cortical microcircuit model. Front. Neurosci. 12:291 10.3389/fnins.2018.00291 29875620 PMC5974216

[B91] Vassanelli S. Mahmud M. (2016). Trends and challenges in neuroengineering: toward “Intelligent” neuroprostheses through Brain-“BrainInspiredSystems” communication. Front. Neurosci. 10:438 10.3389/fnins.2016.00438 27721741 PMC5034009

[B92] Vogelstein R. Mallik U. Cauwenberghs G. (2004). Silicon spike-based synaptic array and address-event transceiver, IEEE International Symposium on Circuits and Systems (Vancouver, BC).

[B93] Walter A. Murguialday A. Spüler M. Naros G. Leão M. Gharabaghi A.. (2012). Coupling BCI and cortical stimulation for brain-state-dependent stimulation: methods for spectral estimation in the presence of stimulation after-effects. Front. Neural Circuits 6:87 10.3389/fncir.2012.00087 23162436 PMC3499764

[B94] Wang R. Cohen G. Stiefel K. Hamilton T. Tapson J. Van Schaik A. (2013). An FPGA implementation of a polychronous spiking neural network with delay adaptation. Front. Neurosci. 7, 1–14 10.3389/fnins.2013.00014 23408739 PMC3570898

[B95] Webb A. C. Burns B. D. (1976). The effects of changing levels of arousal on the spontaneous activity of cortical neurones I. Sleep and wakefulness. Proc. R. Soc. Lond. B Biol. Sci. 194, 225–237. 10.1098/rspb.1976.0075 11487

[B96] Yuan N. Fu Z. Zhang H. Piao L. Xoplaki E. Luterbacher J. (2015). Detrented partial-cross-correlation analysis: a new method for analyzing correlations in complex system. Sci. Rep. 6:27707 10.1038/srep27707 PMC4311241 25634341

[B97] Zhang N. Ding S. Zhang J. Xue Y. (2018). An overview on restricted boltzmann machines. Neurocomputing 275, 1186–1199. 10.1016/j.neucom.2017.09.065

